# Development of Gluten‐Free Extruded Snack Containing Lentil Flour and Evaluation of Extrusion Process Conditions on Quality Properties

**DOI:** 10.1002/fsn3.70663

**Published:** 2025-07-28

**Authors:** Buse Ozlem Esen, Neslihan Bozdogan, Lara Mina Kutlar, Seher Kumcuoglu

**Affiliations:** ^1^ Department of Food Engineering, Faculty of Engineering Ege University Bornova Izmir Turkey; ^2^ Department of Agronomy, Food, Natural Resources Animals and Environment (DAFNAE) University of Padua Legnaro Italy

**Keywords:** carrot pomace powder, extrusion, gluten‐free extruded snack, lentil flour, pasting properties, SEM

## Abstract

Increased knowledge about celiac disease and health concerns has generated increased demand for nutritious gluten‐free snacks; however, most extruded gluten‐free foods remain low in protein and fiber. This study aimed to develop gluten‐free extruded snacks with improved nutritional and functional properties using lentil flour (LF) and to investigate the impact of extrusion conditions on the properties of extruded snacks. Extruded snacks were produced using a corotating twin‐screw extruder with different ratios of LF to corn grits (CG) (0:75, 25:50, 50:25, and 75:0), with fixed amounts of tomato powder, potato flour, and carrot pomace powder. Two feed moisture (FM) levels (16% and 18%) and three barrel temperatures (BT) (135°C, 150°C, and 165°C) were also investigated. The pasting properties of the flour blends and functional, morphological, sensory, and physicochemical characteristics of the extrudates were assessed. The variation in pasting properties of blends was significant; higher CG content increased paste temperature from 67.0°C to 76.6°C, while LF addition reduced it. Increasing LF and FM negatively influenced the expansion ratio (ER) (3.55–1.55) whereas positively affected apparent density (AD) (0.43–2.63 g/cm^3^). BT had the opposite effect on AD, with higher BT leading to reduced density. LF and FM significantly affected the texture properties; the hardness increased from 86.72 N to 399.63 N, and the crispness decreased with increasing LF and FM. LF addition increased the a* value, whereas higher BT reduced redness. Additionally, LF exhibited the highest water solubility index (32.53%), while CG had the highest water absorption index (5.39 g/100 g). SEM analysis revealed denser and less expanded formations at higher LF levels. These findings suggest that lentil flour can improve the nutritional and functional properties of gluten‐free extruded snacks when the extrusion conditions are adequately controlled. This could offer a viable option for health‐conscious consumers and those with gluten intolerance.

## Graphical abstract

This study investigates the physicochemical, functional, morphological, and sensory properties of gluten‐free extruded snacks made from varying proportions of lentil flour (LF) and corn grits (CG) with carrot pomace powder, potato flour, and tomato powder in fixed levels. The results indicate that increasing LF levels raise protein content but reduce expansion, enhance hardness, and alter pasting properties due to starch–protein interaction. Extrusion conditions significantly impact product texture, density, and solubility, which provides valuable information to optimize gluten‐free snack food formulations with improved nutritional and structural qualities.

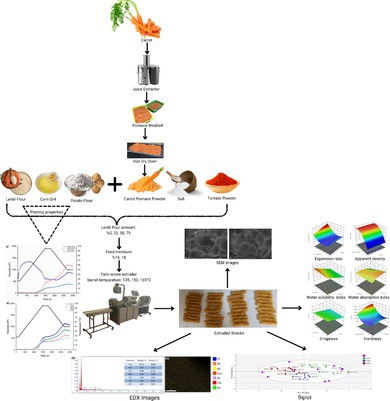

## 1 Introduction

In recent years, gluten‐free diets have gained heightened interest, especially with the rising prevalence of celiac disease, gluten sensitivity, and wheat allergies (Aljada et al. 2021). Celiac disease is an immune disorder triggered by gluten ingestion, which causes damage to the small intestine and impaired nutrient absorption (Igual et al. 2024). Currently, the only effective treatment is the strict and lifelong adherence to a gluten‐free diet. Beyond those with diagnosed conditions, many consumers also choose gluten‐free products for their perceived health benefits; hence, the demand for nutritious and functional gluten‐free alternatives has become one of the leading research focuses (Aljada et al. 2021).

Apart from their health significance, gluten‐free foods are a rapidly expanding part of the international food market. Recent estimates place the global gluten‐free food market size at USD 14.1 billion in 2025 and USD 33 billion in 2034 at a Compound Annual Growth Rate of 9.9% over the forecast period (Global Market Insights 2025). This growing economic significance highlights the need to create innovative, nutritionally better gluten‐free foods with high consumer acceptability.

Despite this demand, most commercially available gluten‐free snacks are made from refined flours and starches and generally have low fiber and protein content (Gumul et al. 2023). This nutritional deficiency comprises one of the significant challenges when developing gluten‐free snacks that are health‐promoting yet sensorially acceptable (Aljada et al. 2021). In order to make up for gluten‐free formulations usual nutritional deficiencies, recent studies have highlighted the need to utilize nutrient‐dense, functional additives. Incorporation of pulses, vegetables, and some industrial by‐products in extruded snacks is one promising approach; such ingredients will raise the fiber and protein levels and improve the nutritional profile of the products (Igual et al. 2024).

Due to its high protein content, dietary fiber, and slowly digestible carbohydrate content, which not only improve nutritional value but also adds to functional and textural qualities, lentil flour (LF) was chosen as the primary ingredient in this case (Morales et al. 2015; Ahmed et al. 2016). LF's high protein and fiber content, however, may have a negative impact on the crispness and expansion of extruded goods. Secondary additives like tomato powder (TP), potato flour (PF), and carrot pomace powder (CPP) were added to overcome nutritional drawbacks and increase product appeal. Rich in insoluble fiber, CPP enhances nutrition and maintains texture (Ahmad et al. 2016; Kırbaş et al. 2019). While TP contributes natural color, antioxidants, and flavor to further improve sensory quality, PF was selected due to its starch content and plasticizing impact, which can enable expansion and structure creation during extrusion (Nazlım and Tuncel 2018). These components were combined to provide a synergistic composition that offers a gluten‐free snack that is both nutrient‐dense and palatable to the senses. Considering the common fiber and protein deficiencies in conventional gluten‐free formulations, LF, PF, CPP, TP, and other ingredients may well provide an excellent opportunity to add value to the development of new extruded snacks (Ahmed et al. 2016).

Extrusion technology, a brief but intense thermomechanical process, plays a vital role in this context. It makes gluten‐free snacks more digestible and extends the shelf life, thus providing proper textural properties (Pasqualone et al. 2021). This technology improves nutritional bioavailability at a low cost and is industrially scalable (Maskan and Altan 2012).

Despite these benefits, there is still a knowledge gap in studies to identify appropriate extrusion conditions (barrel temperature, screw speed, and feed moisture) to improve the structural, nutritional, and sensory properties of gluten‐free extruded snacks (Ali et al. 2024). Determination of appropriate extrusion conditions is critical to attaining desired product texture, expansion, crispness, and nutrient retention, which are generally lost in gluten‐free foods. Deviation from ideal processing conditions (either below or above ideal levels) can significantly impact the quality of gluten‐free extruded snacks. For instance, under‐barrel temperature or low screw speed can result in incompleteness of starch gelatinization, poor expansion, and unacceptable texture. Conversely, very high temperature or high moisture content can result in overplasticization of the matrix, which can result in collapsed structure, hardening, and reduced crispness. In both cases, nutrient degradation and reduced consumer acceptability are likely outcomes (Lazou and Krokida 2010; Saeleaw et al. 2012).

Most current research on gluten‐free extruded snacks is primarily interested in the functional roles of ingredients (e.g., pulses, vegetable by‐products) (Gumul et al. 2023; Igual et al. 2020, 2024). Relatively fewer studies have thoroughly investigated how extrusion processing conditions affect the overall quality and texture of extrudates (Pasqualone et al. 2021; Hood‐Niefer and Tyler 2010), and very few have integrated both perspectives.

In contrast to previous studies that have typically examined ingredient functionality or extrusion conditions separately, this study integrates both formulation design and the selection of key processing conditions to evaluate their combined effect on the quality of gluten‐free vegetable‐based snacks using LF. This research addresses this gap by investigating changes in lentil flour incorporation and extrusion operational variables and how they influence the quality attributes of gluten‐free extruded snacks. A systematic experimental approach was used to evaluate the individual and combined effects of these variables. These results contribute to a deeper understanding of the relative impact of formulation and processing conditions on the quality of gluten‐free snacks, in turn providing insights that can be exploited in the formulation of more acceptable nutritious products for celiac patients and people concerned about their health.

## 2 Materials and Methods

### 2.1 Materials

CG (Semolina Azteca Milling Food Inc., Türkiye) (11.84% moisture, 5.40% protein, 0.40% ash, 1.01% oil and particle distribution of grit > 500 μm for 50–65 g, and > 300 μm for 35–50 g per 100 g of grit), LF and PF (Değirmen Inc., Türkiye) (LF: 9.07% moisture, 32.43% protein, 2.41% ash, and 0.97% oil) (PF: 7.72% moisture, 11.75% protein, 4.38% ash and 1.09% oil), TP (Kurucum Food Inc., Türkiye), and salt were employed as raw materials. CPP was produced under laboratory conditions using Nantes ( *Daucus carota* L.) type carrots obtained from a local greengrocer. Boric acid, copper sulfate, potassium sulfate, sulfuric acid, hydrochloric acid, and hexane (Merck) were employed in analyses. Every chemical employed in the study was of analytical quality.

### 2.2 Methods

#### 2.2.1 Obtaining Carrot Pomace Powder

CPP (6.06% moisture, 4.59% protein, 5.80% ash, and 2.10% oil) was obtained from Nantes ( *Daucus carota* L.) type carrots. After cleaning, the carrots were juiced using a juice extractor (Santos SA, Lyon, France). The pomace was washed twice with lukewarm water (40°C) to remove excess sugars and starch, preventing undesirable stickiness and hardness in the final product, and then dried at 65°C until a moisture content of 2%–3% was reached in an oven (Memmert, UF110, Schwabach, Germany) (Kırbaş et al. 2019; Baljeet et al. 2014). The pomace was ground using a grinder (WSG60E, Waring Commercial, Torrington, CT) and sifted through a 355 μm mesh. It was then stored at 25°C in a dark, cool, and dry location until the time of ES production.

#### 2.2.2 Sample Preparation and Extrusion Process

Powder blends (PBs) were prepared by mixing LF and CG in the ratio of LF:CG (0:75, 25:50, 50:25, 75:0) to investigate the effect of LF. Salt (1.5%), PF (15%), CPP (5%), and TP (3.5%) were the flavorings, which summed up to a total of 25% and kept constant. The feed moisture content of every powder blend was adjusted to 16% or 18% wet basis by spraying calculated amounts of distilled water over the dry blends based on their initial moisture content and finally by blending thoroughly. The blends were placed in polyethylene bags and stored overnight at 25°C for uniform distribution of moisture. Before extrusion, the actual moisture content of each blend was confirmed using an infrared moisture analyzer (Radwag, Model: MA 50. R, Poland) and adjusted if there was any discrepancy from the desired one.

Experimental design was formulated with eight different powder blend (PB) formulations prepared by blending different levels of LF (LF: 0%, 25%, 50%, 75%), FM levels (FM: 16% or 18%), and a constant 25% flavoring mixture (composed of PF, CPP, TP, and salt). Corn grits (CG) were incorporated to make it 100% in each formulation. Each of these 8 PBs was extruded at three BT (BT: 135°C, 150°C, and 165°C), resulting in 24 extruded snack (ES) samples. Substitution levels of LF (0%–75%) were selected based on earlier studies that experimented with the incorporation of pulses into gluten‐free foods (Morales et al. 2015; Pasqualone et al. 2021) and preliminary trials for achieving acceptable texture and processability. Feed moisture (FM) levels (16% and 18%) were chosen within the typical range for cereal extrusion, considering literature reports (Lazou and Krokida 2010; Costantini et al. 2021) and machine functioning limitations. Barrel temperatures (135°C, 150°C, 165°C) were chosen to cover a low‐to‐high thermal input gradient that has been found to have significant impacts on expansion and starch conversion during extrusion (Ding et al. 2006; Alam et al. 2016). Temperature control was achieved in the fourth barrel zone and the die zone, which are thermally interrelated during the extrusion process. These zones are crucial for the completion of the final starch gelatinization, melt behavior, and expansion. Since the die zone temperature is greatly affected by the fourth zone setup, this zone presented a chance for us to observe its influence on the product quality directly.

The extrusion process was conducted using a corotating twin‐screw extruder (Feza Machine Co. Ltd., Istanbul, Türkiye) with a 25 mm barrel diameter and length‐to‐diameter (L/D) ratio of 25:1. The screw configuration consisted of conveying and kneading elements for providing homogeneous mix and adequate shear. Shaping was performed with a circular die having an orifice diameter of 3 mm. The extruder possessed a five‐zone barrel temperature system (Zone 1: 60°C, Zone 2: 100°C, Zone 3: 130°C, Zone 4: 135°C, and die zone: either 135°C, 150°C, or 165°C depending on the experimental run), with all zone temperatures set and regulated using the manufacturer‐installed monitoring software. The speed of the screw was maintained at 200 rpm (Lazou and Krokida 2010; Costantini et al. 2021), while the feed rate was set at 7.62 kg/h. Screw speed was fixed at 200 rpm after preliminary trials, and the literature reported this level as being suitable for lentil and corn extrudates (Morales et al. 2015; Lazou and Krokida 2010). In this manner, the study concentrated on the effects of FM, BT, and LF content on product quality. Though screw speed also influences significant physical and functional properties, it was not included as a variable in the current design to avoid making the factorial design too complicated. A simplified schematic diagram of the extrusion equipment is shown in Appendix S2 to facilitate a clear visualization of equipment layout and process conditions. After extrusion, the extruded snacks were dried at 50°C for 1 h in a laboratory‐scale hot air oven (Memmert, UF110, Schwabach, Germany), then cooled to room temperature (25°C) and stored in plastic polyethylene terephthalate jars.

#### 2.2.3 Pasting Properties

The pasting properties of LF, CG, PF, and PBs were determined using a rheometer with a pasting cell (DHR3, TA Instruments, USA). Samples (2.5 g) and distilled water (22.5 mL) were added to the cell, and the analysis began immediately. The sample‐to‐water ratio was modified from the earlier studies. The temperature profile included holding at 25°C for 1 min, heating to 95°C at a rate of 3°C/min, holding at 95°C for 5 min, cooling to 50°C at a rate of 3°C/min, and then holding at 50°C for 5 min (Darfour et al. 2012; Guha et al. 1998). Pasting temperature (PaT), peak viscosity (PV), trough viscosity (TV), final viscosity (FV), and setback viscosity (SV) were determined from the curves. All measurements were conducted in triplicates, and the mean values of the data were presented.

#### 2.2.4 Proximate Analyses

According to AOAC (2005) procedures, the moisture (Method: 925.10), protein (Method: 930.29), fat (Method: 945.38), and ash (Method: 923.03) content of the CPP, LF, CG, PF, and extruded snacks was performed. All analyses were conducted at least in triplicate.

#### 2.2.5 Bulk Density, Expansion Ratio, and Apparent Density

To determine the bulk density (BD) of raw materials, 20 g of each of CPP, LF, CG, and PF was weighed into a 100 mL graduated beaker and tapped gently until the sample level stabilized. BD was calculated as the sample weight per unit volume (g/mL) (Du et al. 2014).

The expansion ratio (ER) of the extruded snacks was calculated as the ratio of the extrudate diameter to the die diameter (Equation 1) using 10 randomly selected 5 cm long samples (Thymi et al. 2005).

(1) $$\mathrm{ER}=d_{\text{extrudate}}/d_{\mathrm{die}}$$

The apparent density (AD) of the extruded snacks was calculated using the formula given in Equation (2) (Thymi et al. 2005), where *D* is the diameter of the extrudate (cm), and *L* represents the total length of extrudate per unit mass (cm/g).

(2) $$\mathrm{AD}\;\left(\frac{g}{{\mathrm{cm}}^{3}}\right)=\frac{4}{\pi D^{2}L}$$

#### 2.2.6 Textural Properties

The hardness (H) and crispiness (CR) of the extruded snacks were determined using a TA‐XT2i Texture Analyzer (StableMicrosystems, Surrey, UK) equipped with a five‐blade Kramer cutting cell. Texture analysis was performed at a pretest speed of 1 mm/s, test speed of 2 mm/s, and post‐test speed of 10 mm/s, respectively. These speed values were chosen based on previous studies to measure forces during compression and shearing accurately (Oliveira et al. 2017). In each test, at least 10 extrudate samples were analyzed, and measurements were done in triplicate to ensure reproducibility. The greatest force required to shatter the sample was denoted by H, and the total number of force peaks seen along the curve was known as CR (Dogan and Kokini 2007).

#### 2.2.7 Color Analysis

Color measurements of CPP, LF, CG, PF, and extruded snacks were performed using a Konica Minolta spectrophotometer (CM‐700D, Konica Minolta Sensing, Japan) based on the Hunter color system. Prior to measurement, all samples, including ESs, were crushed to a fine powder and gently spread in a Petri dish to form a smooth surface. Each sample was measured at not less than 12 random points for L* (lightness/darkness), a* (redness/greenness), and b* (yellowness/blueness) color values in the CIELAB color space.

#### 2.2.8 Functional Properties

The water solubility index (WSI) (Equation 3) and water absorption index (WAI) (Equation 4) parameters of CPP were measured using a distinct method from other raw materials and extruded snacks due to its different physicochemical characteristics, such as high fiber content and unique hydration behavior.

For CPP, 0.5 g of sample was mixed with 19.5 mL of distilled water and stirred at 200 rpm for 24 h (Schmid et al. 2020). For the WSI, the mixture was centrifuged at 4600 *g* for 50 min at 20°C (Hettich Universal 320R cooled, Germany), and the supernatant was collected and dried at 105°C until a constant weight was achieved. For the WAI, the stirred samples were kept at room temperature (25°C) for 3 h before separating the supernatant. The wet pellet and the pellet dried at 105°C were weighed.

To determine the WSI and WAI of LF, PF, CG, PBs, and ESs, specific sample‐to‐water ratios and processing conditions were used. For WSI analysis, 0.5 g of each sample and 10 mL of distilled water (Espinosa‐Ramírez et al. 2021) were stirred at 300 rpm for 1 h. The well‐mixed samples were centrifuged at 4500 *g* for 15 min, and the supernatant was collected and dried at 105°C until constant weight. For WAI measurement, 2.5 g of each sample and 30 mL of distilled water (Du et al. 2014) were mixed and incubated in a water bath at 70°C for 30 min. After cooling to room temperature (25°C), the mixtures were centrifuged at 3000 *g* for 20 min, and the supernatant was carefully separated. The weights of the wet pellet and the pellet dried at 105°C were measured.

(3) $$\mathrm{WSI}\;\left(\%\right)=\frac{m_{\text{supernatant},\text{dried}}}{m_{\text{powder}}}\times 100$$

(4) $$\mathrm{WAI}\;\left(\%\right)=\frac{m_{\text{pellet},\mathrm{wet}}-m_{\text{pellet},\text{dried}}}{m_{\text{pellet},\text{dried}}}$$

Although oil absorption capacity (OAC) is commonly considered a final food mouthfeel‐stabbing parameter, its determination in raw materials is significant to understanding ingredient functionality, formulation behavior, and potential impact on the food texture of processed food. Since ESs have undergone structural change during extrusion, OAC was researched only in raw material to find out their oil‐binding capacity prior to processing. To determine the OAC of CPP, LF, CG, PF, and PBs, 10 mL of corn oil was added to 1 g of each specimen. CPP samples were kept at room temperature (25°C) for 30 min (Baljeet et al. 2014), then centrifuged at 2000 *g* for 10 min. The other samples were centrifuged at 2000 *g* for 30 min and then kept at room temperature for 1 h (Darfour et al. 2012). The volumes of free oil were recorded, and the OAC was expressed as a percentage of the sample weight.

#### 2.2.9 Scanning Electron Microscopy (SEM) and Energy‐Dispersive X‐Ray (EDX) Analysis

The morphological properties of the extruded snacks were examined using a scanning electron microscope (Thermo Scientific Apreo S, Waltham, MA, USA). Prior to the analysis, extruded snacks were cut into 5–10 mm thick pieces (Philipp et al. 2017), frozen at −40°C, and then lyophilized (Martin Christ Alpha 1‐2 LD plus, Germany). SEM images were captured at magnifications of 150×, 200×, 250×, 500×, and 1000× using accelerated voltages of 10–20 kV. Each sample was analyzed in triplicate to ensure reproducibility. The distribution spectra of elements C, N, O, Na, P, Cl, and K were obtained through energy‐dispersive X‐ray (EDX) analysis, and measurements were performed on at least three different regions per sample.

#### 2.2.10 Sensory Analysis

Sensory evaluations of extruded snacks were carried out with 15 semitrained panelists, selected from graduate students and faculty members of the Ege University Food Engineering Department. With a mean age of approximately 25, the panelists ranged between 22 and 30 years old. Two preliminary sessions trained the panelists, where they were introduced to the evaluation criteria and trained with reference samples representing different intensity levels for texture (crispiness and hardness) and appearance (color). Panelists received training in the training session on the usage of the nine‐point hedonic scale and were also given sample exercises for achieving a consistent score. Appearance (color), texture (CR, H), and overall acceptability of extruded snacks were rated on the basis of the nine‐point hedonic scale (e.g., 9 = liked extremely, 1 = not at all).

#### 2.2.11 Statistical Analysis

All experiments were performed in duplicates, and for each replicate, measurements were conducted in triplicates. Values are expressed as mean ± standard deviation. SPSS Version 22.0 software (SPSS Inc., Chicago, IL) was utilized to compare experimental data statistically. Physicochemical and textural properties such as expansion ratio, hardness, crispness, and apparent density of the extruded samples were subjected to a three‐way ANOVA to study the main effects and interaction effects among barrel temperature, feed moisture, and lentil flour content. The general linear model procedure was implemented, considering BT, FM, and LF as fixed factors. Duncan's multiple‐range test was conducted on all samples irrespective of factor levels when an overall significance was recorded (*p* ≤ 0.05) so as to assign a set of mutually statistically homogeneous groups. Along with the *F*‐values and associated *p* values, partial eta squared (*η* ^2^) values were given to indicate the strength and significance of the effects of each factor for each response variable. Observed power values assessing the reliability of the tests were also calculated. Before the calculation of power, considerations of normality and homogeneity of variance were ensured to validate the use of ANOVA. Analyses were carried out to examine the main factors separately and also across their interaction effects, which would consequently shed some light on either synergistic or antagonistic influences that processing parameters might have on each other. The interdependencies among the dependent variables (ER, AD, WSI, WAI, CR, and H) were investigated using principal component analysis (PCA). The scores plot and loadings plot were complementary plots. Hierarchical clustering on principal components (HCPC) was employed to characterize cluster interactions. This clustering process, based on Ward's method, clustered the snacks according to their Euclidean distance dissimilarities. All the calculations were performed using SIMCA software (Trial Version 14.1, Umetrics, Umeå, Sweden).

To investigate the relationships between instrumental measurements of texture and sensory evaluations, a Pearson correlation analysis in Microsoft Excel (Microsoft Corp., USA) was carried out. Texture parameters, namely hardness or crispiness, as obtained by instrumental measurements, were correlated to sensory attributes like perceived hardness or crispiness. Instrumental and sensory data for each sample were paired to ensure direct comparability. Correlation coefficients (*r*) were calculated, and the significance was tested at a 95% level of confidence (*p* < 0.05). An *r* above 0.7 was considered to indicate a good correlation between instrumental and sensory measurements, while values between 0.5 and 0.7 were considered moderate.

## 3 Results and Discussion

### 3.1 Pasting Properties

**FIGURE 1:**
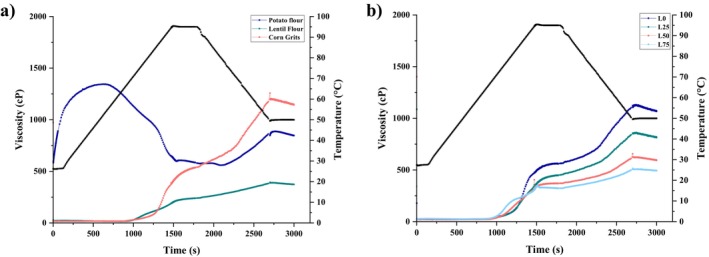
Pasting properties of (a) potato flour (PF), lentil flour (LF), and corn grits (CG) of pasting properties and (b) powder blends (PBs).

The pasting profiles of the raw materials and PBs in excess water are shown in Figure 1, and the parameters PaT, PV, TV, FV, and SV obtained from pasting curves are shown in Table 1. There were statistically significant variances (*p* ≤ 0.05) observed in the pasting properties of the samples. CG, LF, and PF exhibited significantly different pasting characteristics, while the PBs showed similar pasting characteristics to corn grits. CG had a higher PaT than LF, and increasing the level of LF in the PB led to PaT reduction, which can be attributed to diverse causes. Smaller particles present in LF enhance the hydration and lead to earlier gelatinization. Sharma et al. (2023) found that finer gorgon nut flour fractions (< 106 μm) exhibited lower pasting temperature and viscosities because the finer particles retained more water with a higher surface area. Similarly, in our study, increasing LF content caused a reduction in pasting temperature from 72.54°C in the control sample to 66.68°C in the L50 blend. Additionally, the protein content of lentil flour is greater and has been reported to interact with starch and reduce the pasting temperature (Li and Ganjyal 2017). It was observed by Li and Ganjyal (2017) that lentil flours exhibited lower pasting temperatures and peak viscosities compared to pea flours due to their higher protein content that interferes with starch gelatinization. Our results support this finding. The peak viscosity was observed to gradually decrease to 306.537 cP for the L75 blend from 357.600 cP in CG. The soluble fiber content and the inherent starch in lentil flour can be another cause for this reduction in PaT (Ronie and Hasmadi 2022). PF did not exhibit a PaT, which may be attributed to the starch granules that were compromised as a result of the mechanical and thermal pretreatments, such as washing and boiling of potato flour, which destroyed their water absorption ability and swelling, hence preventing the normal pasting behavior of undamaged starch (Nazlım and Tuncel 2018).

**TABLE 1:** Pasting properties and functional of raw materials and powder blends.

Samples	Pasting temperature (PaT) (°C)	Peak viscosity (PV) (cP)	Through viscosity (TV) (cP)	Final viscosity (FV) (cP)	Setback viscosity (SV) (cP)	Water absorption index (WAI) (g/100 g)	Water solubility index (WSI) (g/100 g)	Oil absorption capacity (OAC) (mL/g)
*LF*	67.935 ± 0.32	188.550 ± 0.35	235.850 ± 1.34	371.750 ± 2.19	135.900 ± 0.85	1.439 ± 0.074^a^	27.312 ± 0.646^c^	1.495 ± 0.139^a^
*PF*	—	1345.900 ± 1.56	595.250 ± 14.92	851.150 ± 6.29	255.900 ± 8.63	7.474 ± 0.502^c^	18.764 ± 0.042^b^	1.745 ± 0.070^a^
*CG*	76.575 ± 0.52	357.600 ± 28.28	603.950 ± 108.40	1293.650 ± 206.97	689.700 ± 98.57	2.554 ± 0.121^b^	2.929 ± 0.070^a^	1.585 ± 0.001^a^
*CPP*	—	—	—	—	—	32.599 ± 0.112^d^	17.9736 ± 0.584^b^	2.693 ± 0.136^b^
L75	67.000 ± 0.12^a^	306.537 ± 7.55^a^	327.778 ± 10.54^a^	493.943 ± 8.84^a^	166.165 ± 1.69^a^	3.025 ± 0.091^a^	32.533 ± 1.410^c^	1.196 ± 0.003^a^
L50	66.680 ± 0.30^a^	311.136 ± 5.94^a^	363.357 ± 5.62^b^	583.830 ± 12.50^b^	220.634 ± 6.87^b^	3.370 ± 0.275^a^	23.537 ± 2.055^b^	1.891 ± 0.135^b^
L25	69.265 ± 0.85^b^	330.205 ± 7.81^a^	438.535 ± 7.77^c^	786.417 ± 28.40^c^	348.190 ± 20.62^c^	4.437 ± 0.172^b^	21.081 ± 1.230^b^	2.089 ± 0.133^b^
L0	72.540 ± 0.15^c^	475.950 ± 5.34^b^	567.363 ± 7.11^d^	1082.175 ± 12.87^d^	514.812 ± 5.76^d^	5.394 ± 0.139^c^	10.626 ± 0.119^a^	1.798 ± 0.28^b^

*Note:* *Values with similar superscripts in the column do not differ significantly (*p* < 0.05). **The coding of the samples was expressed to indicate LF (lentil flour), PF (potato flour), CG (corn grits), and CPP (carrot pomace powder). The letter L represents the lentil flour in the powder blends, and the number next to it indicates the amount (%) in the sample. For example, L75 denotes 75% lentil flour amount. ***indicates value not determined.

During the heating and holding phase at peak temperature, CG was observed to increase in viscosity steadily (Figure 1a), indicating starch gelatinization from enhanced molecular reorganization in starch granule amorphous regions under highly controlled thermal and hydration environments. Structure stabilization that renders it harder to degrade granular integrity and create a paste concurs with mechanisms reported for starch‐rich systems (Zhang et al. 2020). In contrast, PBs with LF showed LF content‐modulated viscosity profiles. The L0 blend of predominantly CG with added PF and CPP mirrored CG's steady increase in viscosity, which suggested minimal interference with starch reorganization. Despite that, inhibitory effects were triggered by increasing content of LF: The L25 mixture exhibited retarding viscosity growth, presumably resulting from partial replacement of LF‐sourced proteins for starch with regard to water intake, restricting swelling of granules, and slowing reconstruction of amorphous areas. Increased LF levels further led to plateauing or decreasing viscosity (Table 1 and Figure 1b), which were caused by a high protein and dietary fiber concentration of LF. Similar findings were also reported for lentil flour systems by Li and Ganjyal (2017). These constituents are most likely form physical barriers around starch granules, confining swelling and leaching of amylose, as well as keeping free amylose to preclude network structure upon cooling. These findings stress the antagonist effect of LF to starch, whereby enhanced concentration increasingly discourages the energy‐mediated structural fixation of CG to finally render the system inefficient at constructing viscosity.

The FV value provides information about the stability of the structure formed after cooking and cooling of a starch mixture. Amylose molecules are effective in the formation of the FV. During the cooking process, amylose molecules seeping out of the particles reassociate during the cooling, leading to agglomeration and viscosity increase (Ratnawati et al. 2019). The process, however, may be disrupted by nonstarch solids such as proteins, dietary fiber, and lipids. CG exhibited the highest FV, while LF demonstrated the lowest, which could be linked to the higher protein content. The protein in the LF competed with starch–water molecules and hindered the starch's swelling, inhibiting amylose leaching (Rachman et al. 2021). The interference reduces free amylose availability for network development, resulting in a lower FV. The decrease of FV with the increasing amount of LF in PBs is additional proof of this mechanism. When the content of LF increased, reassociation of amylose was increasingly delayed, and weaker gelation followed. Of additional significance, proteins and lipids can adsorb directly on amylose molecules, preventing their ordering and crosslinking during cooling. This explains the lowering of the viscosity of LF‐added blends, where nonstarch components protect against amylose‐induced retrogradation. The pasting properties of starch significantly influence the textural qualities of the product and the alterations that may occur during storage. In the present study, formulations containing less amylose had a reduced FV, a trend supported by earlier reports showing that amylose content and FV bear a positive relationship (Singh et al. 2006). The FV, being related to the reassociation of amylose chains during cooling, would lower with the reduced matrix‐forming potential. Therefore, the extrudates thus produced in this study had smaller and less stable air cells, indicating that a low level of FV may adversely affect expansion and texture.

### 3.2 Proximate Analyses

The product's hardness, crispness, longevity on the shelf, and sensory qualities are all strongly impacted by its moisture level. Low moisture retains hardness, while high moisture (often > 6%) may reduce crispness (Chikkanna et al. 2020). According to Table 2, the extruded snacks have a moisture content ranging from 5.50% to 9.42%. Extrusion conditions significantly (*p* ≤ 0.05) affected the moisture content. While an increasing FM and LF increased the MC of the final product, an increase in BT decreased it. During extrusion, a snack usually loses moisture at the die outlet, and it may depend on vapor pressure in air cells of the extrudate, temperature, and matrix characteristics, especially water binding, starch transformation, and extensibility of fiber. Ungelatinized starch, insoluble fiber, and proteins with limited film‐forming ability trap water in the structure instead of releasing it at the vapor flashpoint (Igual et al. 2020). The increase in MC with higher LF is probably due to the higher fiber and protein content in LF. Enzymatic activity, nutrient transport, and cellular processes are just a few of the physiological functions that rely on proteins (Maurya and Said 2014). This makes proteins very important from a nutritional viewpoint, and proteins within legumes are vital regarding quality food because their characteristics will benefit the overall functional and nutritional features of products containing food. The protein content of the extruded snacks (Table 2) was significantly (*p* ≤ 0.05) affected by extrusion parameters. With an increase in the LF content, the protein levels increased in the ESs, whereas with an increase in BT and FM, the protein content was reduced. The reduction in protein content with an increase in BT might be due to heat denaturation. Appendix S3 shows the results of the univariate ANOVA performed for PR. The overall model turned out to be significant (*F* (23, 24) = 457.374, *p* < 0.001), accounting for 99.8% of the total variance in PR (*R* ^2^ = 0.998, Adjusted *R* ^2^ = 0.996). This means that the variation in PR was accounted for by practically all variance in the model, which makes the model very strong in describing the relationships among the factors. The independent factor LF had significant effects on PR (*F* (3, 24) = 3455.589, *p* < 0.001) with a very high partial eta squared value (*η* ^2^ = 0.998); hence, LF explained almost all the variance due to independent variables. Other factors of BT and FM also had statistically significant effects on PR (*F* (2, 24) = 39.668, *p* < 0.001, *η* ^2^ = 0.768 and *F* (1, 24) = 33.994, *p* < 0.001, *η* ^2^ = 0.586, respectively). However, both had smaller effect sizes compared with LF, thus further emphasizing that the primary factor affecting PR is LF, while BT and FM produce secondary effects. Regarding the interaction effect, the interaction effect between LF and FM was significant (*F* (3, 24) = 5.306, *p* = 0.006, *η* ^2^ = 0.399), implying that the effect of LF on PR depends on the level of feed moisture. Although the interaction between LF and BT has not been statistically significant (*p* = 0.090), this might be a noteworthy interaction that deserves further investigation due to the moderate effect size (partial *η* ^2^ = 0.345) describing its practical impact. Furthermore, the interaction between BT and FM, as well as the triple interaction between the three factors, did not reach significance (*p* = 0.133 and 0.397, respectively), suggesting a lack of true synergism or antagonism when paired in different combinations of two or all three.

**TABLE 2:** Physicochemical and functional properties of extruded snacks.

Sample	Moisture (%)	Water activity (*a*_W_)	Protein content (%)	WAI (g/100 g)	WSI (g/100 g)	ER	AD (g/cm^3^)	CR	H (N)
L0M16T135	6.282 ± 0.063^c,d,e,f^	0.336 ± 0.006^b,c^	8.420 ± 0.370^b^	6.145 ± 0.348^h,i^	69.445 ± 4.601^g^	3.550 ± 0.169^m^	0.436 ± 0.022^b^	88.500 ± 8.211^m^	99.495 ± 8.093^a,b,c^
L0M16T150	5.994 ± 0.087^b,c,d^	0.478 ± 0.037^f,g^	8.248 ± 0.370^b^	5.560 ± 0.320^f,g,h^	68.435 ± 4.109^g^	3.752 ± 0.113^n^	0.078 ± 0.004^a^	68.875 ± 5.384^k^	86.720 ± 7.364^a^
L0M16T165	6.808 ± 0.029^g^	0.484 ± 0.013^i,j^	7.210 ± 0.366^a^	4.438 ± 0.276^c^	65.651 ± 3.307^f,g^	2.725 ± 0.06^e^	0.613 ± 0.021^c,d^	38.625 ± 3.462^d,e^	111.799 ± 8.707^b,c,d^
L0M18T135	7.299 ± 0.067^h^	0.385 ± 0.002^d^	8.171 ± 0.493^b^	3.375 ± 0.08^b^	66.856 ± 3.637^f,g^	3.073 ± 0.130^h,i^	0.843 ± 0.087^f^	66.429 ± 4.541^j,k^	201.083 ± 20.103^h^
L0M18T150	8.916 ± 0.288^k^	0.545 ± 0.007^l^	7.826 ± 0.001^a,b^	5.799 ± 0.225^g,h,i^	64.378 ± 0.091^f,g^	3.106 ± 0.166^i,j^	0.121 ± 0.004^a^	48.000 ± 3.697^g,h^	138.942 ± 11.253^e,f,g^
L0M18T165	6.306 ± 0.094^c,d,e,f^	0.296 ± 0.003^a^	7.134 ± 0.247^a^	3.689 ± 0.368^b^	53.135 ± 4.084^b,c^	2.283 ± 0.073^d^	1.010 ± 0.051^h^	36.500 ± 3.450^c,d^	196.887 ± 15.176^h^
L25M16T135	6.410 ± 0.622^e,f^	0.315 ± 0.005^a,b^	12.386 ± 0.301^d^	3.689 ± 0.368^b^	53.135 ± 4.084^b,c^	3.474 ± 0.151^m^	0.551 ± 0.021^c^	73.875 ± 6.105^l^	98.268 ± 3.942^a,b,c^
L25M16T150	6.071 ± 0.176^b,c,d,e^	0.411 ± 0.018^e,f^	12.130 ± 0.303^c,d^	4.396 ± 0.419^c^	69.186 ± 3.628^g^	3.542 ± 0.110^m^	0.085 ± 0.005^a^	60.125 ± 5.055^i^	93.897 ± 8.369^a,b^
L25M16T165	5.914 ± 0.196^b,c^	0.349 ± 0.005^c^	11.704 ± 0.307^c,d^	5.517 ± 0.014^f,g,h^	66.157 ± 0.321^f,g^	2.134 ± 0.112^c^	0.648 ± 0.037^d,e^	23.600 ± 2.302^b^	214.633 ± 20.157^h,i^
L25M18T135	9.429 ± 0.089^l^	0.553 ± 0.003^l,m^	11.970 ± 0.306^c,d^	5.783 ± 0.221^g,h,i^	65.232 ± 0.929^f,g^	2.723 ± 0.089^e^	0.917 ± 0.051^g^	64.800 ± 6.140^j,k^	243.336 ± 37.732^j^
L25M18T150	6.139 ± 0.241^c,d,e^	0.467 ± 0.009^h,i,j^	11.577 ± 0.119^c,d^	4.873 ± 0.287^c,d,e,f^	56.525 ± 0.914^b,c,d,e^	3.261 ± 0.166^k,l^	0.107 ± 0.009^a^	46.125 ± 2.295^f,g^	117.501 ± 10.408^c,d^
L25M18T165	7.622 ± 0.082^h,i^	0.409 ± 0.002^e^	11.230 ± 0.122^c^	4.760 ± 0.472^c,d,e^	49.941 ± 3.652^a,b^	1.833 ± 0.06^b^	1.625 ± 0.089^i^	22.500 ± 2.082^b^	332.388 ± 20.581^k^
L50M16T135	7.447 ± 0.127^h^	0.440 ± 0.001^f,g^	21.047 ± 0.066^g,h^	4.760 ± 0.472^c,d,e^	49.941 ± 3.652^a,b^	3.346 ± 0.162^l^	0.556 ± 0.025^c^	62.600 ± 2.191^i,j^	110.441 ± 13.357^b,c,d^
L50M16T150	5.502 ± 0.109^a^	0.376 ± 0.010^d^	20.978 ± 0.175^g,h^	4.930 ± 0.100^c,d,e,f^	59.749 ± 5.663^c,d,e,f^	3.193 ± 0.098^j,k^	0.109 ± 0.006^a^	44.500 ± 2.777^f,g^	123.429 ± 10.141^d,e^
L50M16T165	5.715 ± 0.091^a,b^	0.341 ± 0.004^c^	19.770 ± 0.665^e,f^	6.340 ± 0.110^i^	50.788 ± 2.002^a,b^	2.716 ± 0.097^e^	0.701 ± 0.062^e^	20.000 ± 2.000^b^	214.251 ± 22.309^h,i^
L50M18T135	7.917 ± 0.056^i,j^	0.456 ± 0.007^g,h^	20.658 ± 0.675^f,g^	4.459 ± 0.156^c^	52.338 ± 3.404^b^	2.925 ± 0.134^f,g^	0.949 ± 0.077^g,h^	50.833 ± 2.639^h^	200.879 ± 14.933^h^
L50M18T150	6.335 ± 0.213^d,e,f^	0.481 ± 0.012^j^	20.099 ± 0.124^f,g^	4.872 ± 0.289^c,d,e,f^	62.465 ± 0.353^e,f,g^	3.097 ± 0.216^i,j^	0.119 ± 0.005^a^	44.429 ± 2.637^f,g^	125.582 ± 8.308^d,e,f^
L50M18T165	6.219 ± 0.106^c,d,e^	0.377 ± 0.003^d^	19.103 ± 0.059^e^	5.462 ± 0.386^e,f,g,h^	44.332 ± 2.448^a^	1.479 ± 0.063^a^	2.633 ± 0.137^k^	12.600 ± 1.817^a^	399.627 ± 31.952^m^
L75M16T135	6.292 ± 0.144^c,d,e,f^	0.410 ± 0.006^e^	25.231 ± 0.105^k^	5.462 ± 0.386^e,f,g,h^	44.332 ± 2.448^a^	3.243 ± 0.117^k,l^	0.551 ± 0.019^c^	45.143 ± 2.795^f,g^	103.417 ± 7.464^a,b,c^
L75M16T150	6.649 ± 0.084^f,g^	0.462 ± 0.009^g,h,i^	25.246 ± 0.109^k^	4.436 ± 0.203^c^	50.338 ± 2.458^a,b^	2.974 ± 0.105^g,h^	0.128 ± 0.007^a^	42.000 ± 4.106^e,f^	144.178 ± 11.734^f,g^
L75M16T165	6.167 ± 0.37^c,d,e^	0.385 ± 0.02^d^	23.907 ± 0.561^i,j^	4.932 ± 0.073^c,d,e,f^	52.287 ± 3.955^b^	1.556 ± 0.072^a^	2.140 ± 0.091^j^	13.250 ± 1.282^a^	222.036 ± 21.442^i^
L75M18T135	8.040 ± 0.209^j^	0.515 ± 0.012^k^	24.781 ± 0.553^j,k^	5.427 ± 0.218^e,f,g^	49.544 ± 1.457^a,b^	2.934 ± 0.088^f,g^	0.982 ± 0.065^g,h^	45.333 ± 3.011^f,g^	221.989 ± 21.078^i^
L75M18T150	8.046 ± 0.223^j^	0.603 ± 0.038^m^	23.084 ± 0.622^i^	4.643 ± 0.458^c,d^	54.150 ± 4.526^b,c,d^	2.848 ± 0.167^f^	0.131 ± 0.005^a^	33.875 ± 2.475^c^	156.410 ± 11.660^g^
L75M18T165	6.182 ± 0.061^c,d,e^	0.410 ± 0.002^e^	21.780 ± 1.237^h^	2.743 ± 0.263^a^	52.191 ± 1.031^b^	1.552 ± 0.053^a^	2.586 ± 0.142^k^	11.875 ± 1.126^a^	380.068 ± 30.467^l^

*Note:* *Values with similar superscripts in the column do not differ significantly (*p* < 0.05). **The coding of the samples was expressed to indicate LF, FM content, and extruder BT. The numbers after the letter L indicate the LF ratio, the numbers after the letter M indicate the FM ratio, and the numbers after the letter T indicate the extruder BT. For example, code L75M16T165 indicates a product produced at 75% LF, 16% FM, and an extruder BT of 165°C.

Water activity (*a*_W_) values of the extruded snacks ranged from 0.296 to 0.603, as presented in Table 2. In most of the samples, *a*_W_ was under the microbial stability threshold of 0.6, which is an indicator of good shelf stability. Higher FM had the tendency to produce higher *a*_W_ values in most cases, while higher BT had the consequence of decreasing *a*_W_ by means of a higher loss of water. LF content influence was formulation‐dependent; higher LF increased *a*_W_ in some samples through water retention within the dense network of proteins and fibers. The minimum *a*_W_ was in L0M18T165 (0.296), while the maximum *a*_W_ (0.603) was in L75M18T150. These results highlight the effect of both processing temperature and formulation on moisture attributes and shelf‐life potential.

### 3.3 Bulk Density, Expansion Ratio, and Apparent Density

BD is essential in determining the physical characteristics and quality of ESs, influencing properties like apparent density, porosity, hardness, and expansion ratio (Hood‐Niefer and Tyler 2010). Among the samples, CG exhibited the highest BD at 1.026 g/mL, whereas CPP had the lowest at 0.422 g/mL, with LF and PF showing similar values of 0.691 and 0.690 g/mL, respectively. The lower BD of CPP might be because of its lower protein and higher ash content, which may have made the mass lighter, hence suggesting its lower BD (Ahmed et al. 2016). BD is mainly affected by particle size (Ahmed et al. 2016), with CG having larger particles and hence a higher BD.

**FIGURE 2:**
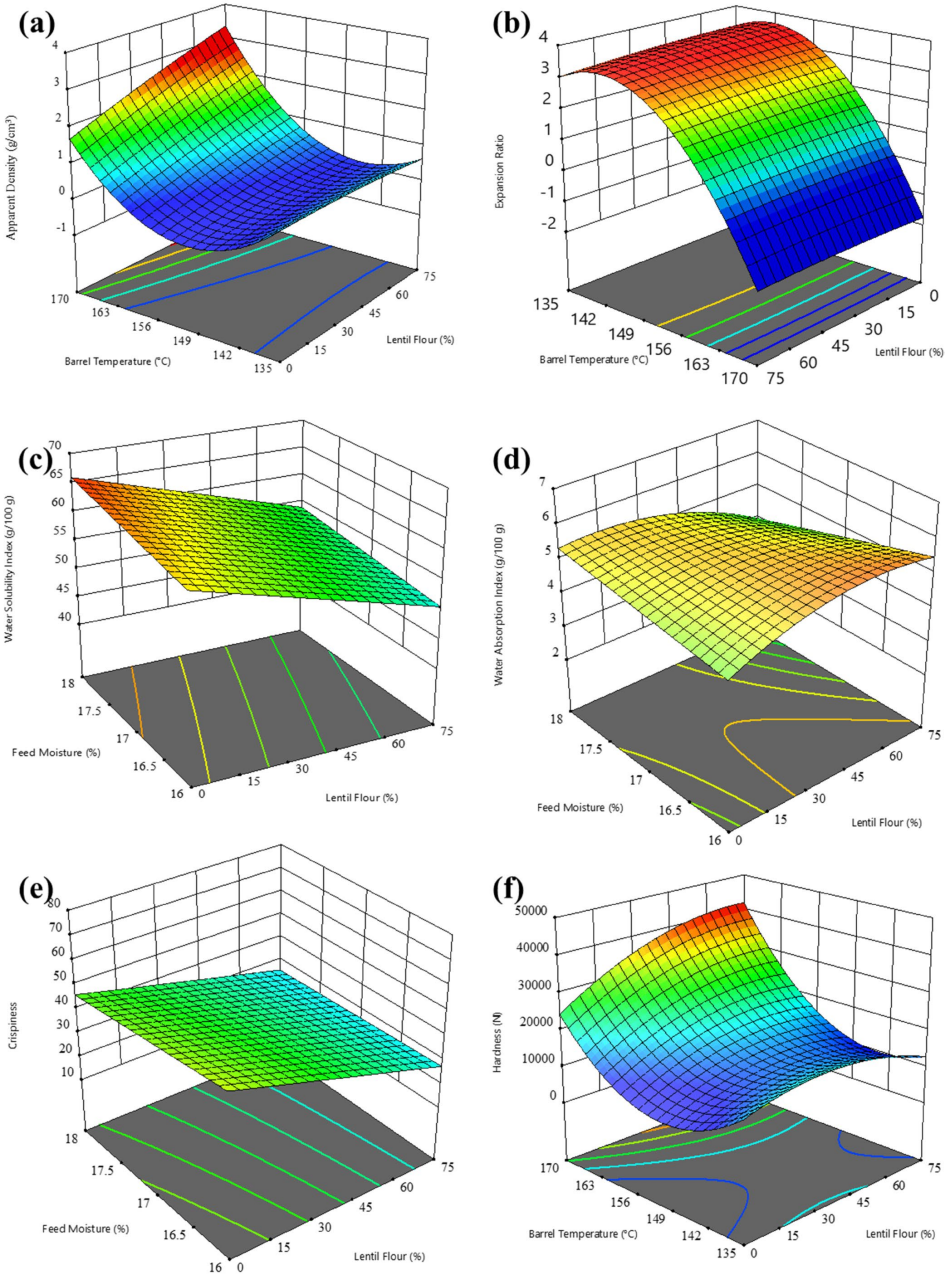
3D surface plots showing effects of feed moisture, and extruder barrel temperature on (a) expansion ratio, (b) apparent density, (c) water solubility index, (d) water absorption index, (e) crispness, and (f) hardness of lentil flour extrudate snacks.

A number of variables, including screw speed, die design, extrusion temperature, feed moisture, and raw material composition, affect the expansion ratio (Costantini et al. 2021). FM, BT, and LF content significantly (*p* ≤ 0.05) influence the ER and AD of the extruded snacks (Table 2, Appendix S3). The three‐way ANOVA results for the dependent variable, ER, show that the overall model was highly significant (*F* (23, 24) = 291.710, *p* < 0.001), with a remarkably high *R*‐square value (*R* ^2^ = 0.969; adjusted *R* ^2^ = 0.965). This means the independent variables (BT, FM, LF, and the interaction effects) explain about 96.9% of the total variation occurring in the expansion ratio. The values of the observed power were very high (1.000 for all significant terms), confirming the reliability of the model. Among the main effects involved, BT was the most significant factor affecting the ER, with a very significant effect (*F* (2, 24) = 2353.336, *p* < 0.001, partial *η* ^2^ = 0.956). The FM also significantly affected ER (*F* (1, 24) = 711.839, *p* < 0.001, partial *η* ^2^ = 0.767). Furthermore, LF is considered significantly different for ER (*F* (3, 24) = 210.467, *p* < 0.001, partial *η* ^2^ = 0.745). ER increased with a decrease in FM, while it decreased with the increase of BT and LF content (Figure 2b). Higher feed moisture during extrusion affects amylopectin structure, reducing melt elasticity, which decreases expansion and increases extrudate density (Lazou and Krokida 2010). The reduced ER with increased LF content can be explained by the protein content in LF that reduces shear, hence causing a reduction of die pressure, translating into a lower pressure difference between the extruder and the atmospheric environment, as earlier suggested by Hood‐Niefer and Tyler (2010). Snacks tend to have a high expansion ratio, showing the inflation of the product correctly, which influences crunchiness and other sensory qualities. Three‐way ANOVA results also indicated that two‐way interaction effects were significant. The BT × FM interaction (*F* (2, 24) = 18.541, *p* < 0.001, partial *η* ^2^ = 0.147) suggests that the effect of BT on expansion changed with feed moisture levels. Similarly, the BT × LF interaction (*F* (6, 24) = 41.614, *p* < 0.001, partial *η* ^2^ = 0.536) pointed out that barrel temperature had a more variable effect depending on the LF content involved. Although the FM × LF interaction also has statistically significant interaction (*F* (3, 24) = 37.250, *p* < 0.001, partial *η* ^2^ = 0.341), it implies that the influence of moisture content on expansion was different for various LF contents.

Results showed that the BT, FM, and amount of LF have a significant effect (*p* ≤ 0.05) on AD. Factorial ANOVA results for AD displayed that the general effect of the model was highly significant (*F* (24, 24) = 1023.818, *p* < 0.001), with a very high proportion of variance of the AD explained by the model (*R* ^2^ = 0.995; adjusted *R* ^2^ = 0.994). This means that BT, FM, LF, and their interactions accounted for 99.5% of the total variation in AD. The observed powers for all significant effects were all 1.000, which further supports the reliability and sensitivity of the analysis. The main effect of BT had the highest effect on AD (*F* (2, 24) = 4160.447, *p* < 0.001, partial *η* ^2^ = 0.984), indicating that changes in temperature or thermal energy during extrusion significantly modified the AD of the extrudates. When the BT increased from 135°C to 150°C, the AD decreased. In comparison, AD increased as the BT increased from 150°C up to 165°C (Figure 2a). Higher extrusion temperatures lead to increased vapor pressure, causing bubble expansion and a less dense structure (Lazou and Krokida 2010). FM had a highly significant effect on AD (*F* (1, 24) = 887.144, *p* < 0.001, partial *η* ^2^ = 0.870). FM was linearly correlated with AD, affecting amylopectin structure in CG and LF, reducing viscosity. High moisture decreases friction between the melt and screw, hindering starch gelatinization, which decreases expansion and increases AD (Bisharat et al. 2013). These findings show that BT and FM significantly affect bulk density. Lower FM (16%) promoted greater expansion and thus lower density, while higher FM (18%) increased melt viscosity and restricted bubble growth, resulting in greater‐density structures. Similarly, higher BT increased starch gelatinization and vapor pressure, lowering density by promoting puffing at high‐shear conditions. The LF content, however, significantly influenced AD (*F* (3, 24) = 468.413, *p* < 0.001, partial *η* ^2^ = 0.955). An increase in LF resulted in increased AD due to higher protein and fiber, which hindered cell wall rupture, thus reducing bubble expansion (Bisharat et al. 2013). As for all two‐way interactions, they were also found to be statistically significant. BT × FM interaction (*F* (2, 24) = 234.146, *p* < 0.001, partial *η* ^2^ = 0.779) showed that the effects of BT on AD were dependent on FM. Similarly, BT × LF interaction was significant (*F* (6, 24) = 492.997, *p* < 0.001, partial *η* ^2^ = 0.957), whereby the influence of barrel temperature on AD was much dependent on LF. FM × LF interaction was significant for AD (*F* (3, 24) = 19.059, *p* < 0.001, partial *η* ^2^ = 0.301), meaning that the effect of FM on AD depends on the LF content, probably due to the difference in water‐binding capacities and swelling behaviors of the powder blends. Moreover, a statistically significant three‐way interaction among BT, FM, and LF variables was indicated by the analysis, *F* (4, 24) = 29.986, *p* < 0.001, partial *η* ^2^ = 0.474. This entails that the synergistic effects of BT and FM conditions on AD were significantly mediated by the LF, thereby necessitating specialized consideration in individual processing strategies for legume‐based extruded snacks.

Starch gelatinization–protein interference equilibrium regulated ER and AD. ER was greater, and AD was smaller for the low‐LF blends, as seemed characteristic of starch‐dominated systems in which efficient gelatinization stabilizes bubble development through extrusion. On the other hand, starch blends with the highest LF showed lower ER and higher AD, as proteins in LF disrupted starch's ability to form cohesive matrices, producing denser structures. These agree with trends in earlier studies of pasting, where LF inhibition decreased the swelling of starch, which led to reduced structural stability levels. Significantly, with high extrusion temperatures, the degradation of starch further reduces the expanded hardness, proving a balance between solubility and structural resistance.

### 3.4 Textural Properties

Texture is an important physical characteristic of snacks (Dogan and Kokini 2007). The texture of extruded snacks is affected by factors including extrusion parameters, feed composition, expansion ratio, and cellular structure of the final product (Philipp et al. 2017). In the extruded snacks, FM, LF content, and BT had a significant impact (*p* ≤ 0.05) on H and CR (Table 2). High protein content can enhance these properties, affecting the overall texture and crispness of the end product (Philipp et al. 2017).

The ANOVA results of the H are given in Appendix S3. The corrected model was significant (*F* (23, 24) = 197.329, *p* < 0.001), with a very high coefficient of determination (*R* ^2^ = 0.965; adjusted *R* ^2^ = 0.960), signifying that 96.5% of the variability in H has been attributed to the model which means a good fit, with processing variables and their interactions substantially affecting the H of the final product. From the main effects, BT had the greatest effect on hardness (*F* (2, 24) = 889.220, *p* < 0.001, partial *η* ^2^ = 0.914), accounting for 91.4% of the variance due to this factor in the model. FM also significantly influenced (*F* (1, 24) = 683.784, *p* < 0.001, *η* ^2^ = 0.804) hardness of the product, with LF at a close third (*F* (3, 24) = 103.190, *p* < 0.001, *η* ^2^ = 0.812). Results explain that these three factors account for significant individual effects on the hardness of the extruded product. From an interaction standpoint, the BT × FM interaction was highly significant (*F* (2, 24) = 171.813, *p* < 0.001, *η* ^2^ = 0.673), indicating that combined effects of BT and FM levels had a strong influence on hardness. Additionally, those significant interaction effects were found for BT × LF (*F* (6, 24) = 17.809, *p* < 0.001, *η* ^2^ = 0.348) and FM × LF (*F* (3, 24) = 8.226, *p* < 0.001, *η* ^2^ = 0.129) were smaller in magnitude relative to those of the main effects but were not negligible. The three‐way interaction (BT × FM × LF) was significant as well (*F* (2, 24) = 5.027, *p* = 0.008, *η* ^2^ = 0.057), albeit with a quite small effect size. Increasing LF content raises H and decreases CR (Figure 2e,f). In the same way, an increase in FM led to an increase in H, probably due to the excess water in the extrusion process. When water is fed in excess amounts, it causes the starch to overplasticize, which decreases the viscosity and ability of the extruder to dissipate mechanical energy. This results in a dense, hard extrudate with a compressed cellular structure and less crispiness (Carvalho et al. 2010). Excess water forbids bubble formation, creating a denser and harder texture, as mentioned by Saeleaw et al. (2012). These results show that hardness is responsive to individual process factors such as barrel temperature and feed moisture and their interaction effects. The three‐way interaction suggests that the influence of any given factor on hardness depends on at least one other processing factor.

Appendix S3 displays the ANOVA procedure for the dependent variable CR. The overall model was highly significant (*F* (23, 24) = 193.695, *p* < 0.001) with an *R* ^2^ of 0.969 (adjusted *R* ^2^ of 0.964). This shows that the model explained around 96.9% of the variance in CR, implying that the independent variables selected, along with their interactions, accounted for nearly all the observable variation in CR. In the analysis of main effects, BT exerted the most substantial main effect on the CR (*F* (2, 24) = 1321.192, *p* < 0.001, partial *η* ^2^ = 0.949), changes in BT explaining about 95% of the variance attributed to independent factors. LF was another significantly influential factor (*F* (3, 24) = 358.581, *p* < 0.001, *η* ^2^ = 0.884) followed by FM (*F* (1, 24) = 172.664, *p* < 0.001, *η* ^2^ = 0.550), evidencing CR's great sensitivity to all three primary processing parameters with BT and LF being of greater importance. Considering interaction effects, all two‐way and three‐way interactions were statistically significant. In detail, the BT × FM (*F* (2, 24) = 16.485, *p* < 0.001, *η* ^2^ = 0.190), BT × LF (*F* (6, 24) = 10.292, *p* < 0.001, *η* ^2^ = 0.305), and FM × LF (*F* (3, 24) = 18.067, *p* < 0.001, *η* ^2^ = 0.278) interactions carried moderate effect sizes; that is, the different combinations of these processing variables indeed exhibited notable influences upon CR. Furthermore, the BT × FM × LF three‐way interaction proved highly statistically significant (*F* (6, 24) = 10.723, *p* < 0.001, *η* ^2^ = 0.313). As BT and FM increase, the CR of the product decreases due to a reduction in the number of thin cell walls and the shrinkage of air pockets at higher temperatures of extrusion, as cited by Saeleaw et al. (2012).

The optimal moisture level for gluten‐free extruded snacks would generally be between 14% and 18%, depending on raw materials and extrusion parameters (Alam et al. 2016; Lazou and Krokida 2010). Moisture contents of 16% and 18% were evaluated in the present study. It was found that reduced moisture (16%) favored better expansion and crispness, while increased moisture (18%) led to greater product density and hardness. Therefore, 16% feed moisture was more suitable for the manufacture of snacks with sensory acceptability and texture at test levels. The texture profile was largely dominated by starch–protein balance. LF‐free mixtures showed ideal crispness, with moderate hardness being contributed by porous starch‐rich structures. LF‐rich mixtures exhibited much higher hardness and low crispness, corresponding to protein aggregate formation and poor starch plasticity. Such texture modifications reflect pasting behavior, where LF proteins inhibited network structure formation of amylose, resulting in brittle, dense matrices. With intermediate LF concentrations, the partial gelatinization of starch yielded balanced textures but at the cost of impairment relative to starch‐focused controls.

### 3.5 Color Analysis

Color is one of the essential factors that determine consumer preferences. It depends on the variables such as shear force, temperature, feed composition, and duration of extrusion (Costantini et al. 2021). Extrusion conditions and formulations significantly influenced the color values (*p* ≤ 0.05). As the LF content increased, L* and b* values decreased, while the a* value increased (Table 3). This can be attributed to the Maillard reaction resulting from the higher protein content of lentil flour (Costantini et al. 2021). Also, lentil flour naturally contains pigments such as carotenoids and polyphenols that can contribute to color changes. These substances cause an increase in redness (a*) and decrease in lightness (*L*) and yellowness (*b*) during high‐temperature extrusion, where pigment degradation and the Maillard reaction occured simultaneously. Increased temperature positively affected L*, whereas there was an adverse effect on a* and b*. L* was maximum at 165°C, which might be due to the degradation of natural color pigments during high‐temperature extrusion (Yu et al. 2013). The hue angle increased as BT increased but decreased with a rise in FM. With LF increasing, Δ*E* exhibited an increasing trend pattern first and then declined. The higher temperature increased the a* values; such a trend caused an increase in ∆*E* values, probably due to the increased Maillard reactions (Yu et al. 2013).

**TABLE 3:** Color and sensory properties of extruded snacks.

Sample	L* (brightness)	a* (red‐green)	b* (yellow‐blue)	∆*E*	Hue angle°	Appearance	Breakability	Crispness	Hardness	Overall acceptability
L0M16T135	59.176 ± 4.094^k,l^	15.324 ± 1.349^e,f,g^	43.169 ± 2.292^j^	—	70.458 ± 1.324^h^	6.25 ± 2.22^b,c,d^	6.65 ± 1.53^a^	6.70 ± 1.66^a,b^	6.30 ± 1.84^a,b^	6.05 ± 1.73^a,b,c^
L0M16T150	56.276 ± 2.500^i,j^	14.569 ± 1.284^c,d,e^	39.019 ± 1.737^f,g^	—	69.547 ± 1.184^g,h^	6.55 ± 2.28^c,d^	6.65 ± 1.98^a^	6.75 ± 1.65^a,b^	6.15 ± 1.90^a,b^	6.10 ± 1.55^a,b,c^
L0M16T165	62.356 ± 2.386^m,n,o^	13.018 ± 0.711^a^	39.770 ± 1.386^f,g,h^	—	71.879 ± 0.689^ı^	7.10 ± 1.45^d^	6.70 ± 1.69^a^	6.40 ± 1.85^a,b^	6.00 ± 1.78^a,b^	6.30 ± 1.41^b,c^
L0M18T135	52.655 ± 2.558^f,g,h^	17.068 ± 0.975^j,k,l^	43.083 ± 1.880^j^	—	68.391 ± 0.723^e,f^	6.65 ± 1.95^c,d^	6.95 ± 1.82^a^	6.65 ± 2.58^a,b^	6.50 ± 2.24^a,b^	5.60 ± 2.30^a,b,c^
L0M18T150	55.403 ± 3.410^i^	14.057 ± 0.921^b,c^	39.000 ± 2.248^f,g^	—	70.149 ± 1.348^g,h^	6.10 ± 1.99^a,b,c,d^	6.40 ± 1.93^a^	6.80 ± 1.50^a,b^	6.60 ± 1.54^a,b^	6.00 ± 1.83^a,b,c^
L0M18T165	61.091 ± 3.214^l,m,n^	14.279 ± 0.842^b,c,d^	42.644 ± 1.911^i,j^	—	71.491 ± 0.560^ı^	5.65 ± 1.69^a,b,c,d^	6.30 ± 1.75^a^	6.45 ± 1.70^a,b^	5.85 ± 2.11^a,b^	5.60 ± 1.76^a,b,c^
L25M16T135	52.816 ± 3.212^f,g,h^	16.163 ± 1.431^g,h,i,j^	39.608 ± 2.500^f,g,h^	9.076 ± 2.824^c,d^	67.819 ± 1.022^e^	6.60 ± 2.13^c,d^	6.50 ± 2.04^a^	7.00 ± 1.89^a,b^	6.60 ± 1.93^a,b^	6.40 ± 1.81^c^
L25M16T150	54.130 ± 2.441^h,i^	14.425 ± 1.126^b,c,d,e^	36.505 ± 1.845^b,c,d^	5.276 ± 2.428^a,b^	68.456 ± 0.951^e,f^	6.60 ± 2.18^c,d^	6.55 ± 2.39^a^	6.95 ± 1.79^a,b^	6.50 ± 1.96^a,b^	6.25 ± 1.86^b,c^
L25M16T165	63.288 ± 1.836^n,o^	13.513 ± 1.017^a,b^	37.988 ± 2.502^d,e,f^	4.180 ± 2.536^a^	70.423 ± 0.581^h^	5.85 ± 2.03^a,b,c,d^	6.40 ± 1.57^a^	6.20 ± 1.85^a,b^	5.85 ± 2.00^a,b^	5.65 ± 1.49^a,b,c^
L25M18T135	54.030 ± 3.770^h,i^	16.623 ± 1.055^i,j,k^	41.198 ± 2.275^h,i^	5.889 ± 3.784^a,b^	68.015 ± 1.084^e^	6.15 ± 1.81^a,b,c,d^	6.75 ± 2.15^a^	6.95 ± 1.93^a,b^	6.45 ± 2.19^a,b^	5.10 ± 1.97^a,b,c^
L25M18T150	50.902 ± 2.531^d,e,f^	15.135 ± 1.194^d,e,f^	36.483 ± 1.573^b,c,d^	6.416 ± 3.228^a,b^	67.483 ± 1.218^d,e^	6.50 ± 1.85^c,d^	6.70 ± 1.26^a^	6.50 ± 1.57^a,b^	6.00 ± 1.78^a,b^	5.75 ± 1.58^a,b,c^
L25M18T165	58.438 ± 1.663^j,k^	15.223 ± 1.166^d,e,f,g^	38.318 ± 2.519^d,e,f,g^	6.009 ± 2.820^a,b^	68.335 ± 0.838^e,f^	5.75 ± 2.02^a,b,c,d^	6.30 ± 1.84^a^	6.00 ± 1.77^a,b^	6.00 ± 1.75^a,b^	5.70 ± 1.56^a,b,c^
L50M16T135	48.242 ± 4.403^b,c^	16.826 ± 1.046^i,j,k^	36.738 ± 2.529^b,c,d,e^	13.648 ± 5.370^e^	65.326 ± 2.034^b^	6.30 ± 1.80^b,c,d^	6.80 ± 1.76^a^	6.70 ± 1.52^a,b^	6.35 ± 1.69^a,b^	6.00 ± 1.97^a,b,c^
L50M16T150	51.360 ± 1.995^e,f,g^	16.555 ± 1.194^h,i,j,k^	37.901 ± 1.915^c,d,e,f^	6.608 ± 1.775^a,b^	66.420 ± 0.824^c^	6.45 ± 1.88^c,d^	7.05 ± 1.90^a^	7.35 ± 1.50^b^	7.05 ± 1.39	6.30 ± 1.62^b,c^
L50M16T165	53.852 ± 2.225^g,h,i^	16.481 ± 0.677^h,i,j,k^	38.284 ± 1.916^d,e,f,g^	9.608 ± 2.659^c,d^	66.698 ± 0.421^c,d^	5.85 ± 1.87^a,b,c,d^	6.55 ± 1.90^a^	6.55 ± 1.73^a,b^	5.95 ± 2.16^a,b^	5.90 ± 1.74^a,b,c^
L50M18T135	48.511 ± 4.142^b,c,d^	16.123 ± 1.363^g,h,i,j^	37.028 ± 2.547^b,c,d,e^	9.171 ± 3.963^c,d^	66.447 ± 1.696^c^	5.80 ± 2.33^a,b,c,d^	6.55 ± 2.56^a^	6.10 ± 2.75^a,b^	6.75 ± 2.63^b^	4.95 ± 2.70^a,b^
L50M18T150	50.843 ± 2.800^d,e,f^	15.832 ± 1.155^f,g,h,i^	36.034 ± 2.542^b,c^	6.307 ± 2.680^a,b^	66.265 ± 1.100^c^	6.50 ± 1.50^c,d^	6.70 ± 1.45^a^	6.40 ± 1.76^a,b^	6.10 ± 1.80^a,b^	5.95 ± 1.82^a,b,c^
L50M18T165	60.584 ± 2.268^k,l,m^	14.560 ± 0.729^c,d,e^	36.548 ± 1.624^b,c,d,e^	7.637 ± 2.069^b,c^	68.275 ± 0.666^e,f^	4.80 ± 2.21^a,b^	6.15 ± 1.53^a^	5.55 ± 1.64^a^	5.20 ± 2.02^a^	5.25 ± 1.55^a,b,c^
L75M16T135	49.969 ± 2.683^c,d,e^	17.916 ± 1.096^l^	38.423 ± 1.309^d,e,f,g^	11.169 ± 4.404^d^	65.003 ± 1.314^b^	6.00 ± 1.55^a,b,c,d^	6.30 ± 1.49^a^	6.50 ± 1.39^a,b^	6.50 ± 1.36^a,b^	5.65 ± 1.66^a,b,c^
L75M16T150	55.946 ± 2.920^i^	17.243 ± 1.046^k,l^	39.963 ± 2.351^g,h^	5.860 ± 2.665^a,b^	66.653 ± 0.717^c,d^	6.05 ± 2.23^a,b,c,d^	6.45 ± 2.18^a^	6.85 ± 1.81^a,b^	6.55 ± 1.79^a,b^	5.95 ± 1.73^a,b,c^
L75M16T165	63.897 ± 1.518^o^	13.658 ± 0.742^a,b,c^	36.046 ± 1.533^b,c^	4.829 ± 2.200^a^	69.246 ± 0.780^f,g^	5.25 ± 2.31^a,b,c^	5.95 ± 2.46^a^	5.80 ± 2.48^a^	5.70 ± 2.54^a,b^	5.50 ± 2.06^a,b,c^
L75M18T135	44.588 ± 2.154^a^	16.776 ± 0.917^i,j,k^	33.508 ± 1.632^a^	13.854 ± 3.609^e^	63.395 ± 1.122^a^	5.35 ± 2.08^a,b,c^	6.30 ± 2.27^a^	5.80 ± 2.59^a^	6.05 ± 2.48^a,b^	4.75 ± 1.94^a^
L75M18T150	46.889 ± 3.228^a,b^	16.370 ± 0.821^h,i,j,k^	35.319 ± 2.339^b^	10.230 ± 4.338^d^	65.075 ± 1.634^b^	6.05 ± 1.76^a,b,c,d^	6.50 ± 1.50^a^	6.45 ± 2.01^a,b^	6.20 ± 1.85^a,b^	5.75 ± 1.58^a,b,c^
L75M18T165	60.468 ± 2.605^k,l,m^	15.591 ± 0.854^f,g,h^	38.473 ± 1.344^e,f,g^	6.157 ± 2.330^a,b^	67.950 ± 0.578^e^	4.65 ± 2.37^a^	6.30 ± 1.87^a^	5.70 ± 1.78^a^	5.80 ± 2.07^a,b^	5.15 ± 1.30^a,b,c^

*Note:* *Values with similar superscripts in the column do not differ significantly (*p* < 0.05). **The coding of the samples was expressed to indicate LF, FM content, and extruder BT. The numbers after the letter L indicate the LF ratio, the numbers after the letter M indicate the FM ratio, and the numbers after the letter T indicate the extruder BT. For example, code L75M16T165 indicates a product produced at 75% LF, 16% FM, and an extruder BT of 165°C.

### 3.6 Functional Properties

Among all the powders, LF had the highest WSI value, as shown in Table 1. Choi et al. (2012) state that the amount of soluble starch, free polysaccharides, and released granules is represented by WSI, a parameter reflecting starch degradation following excessive water addition post extrusion. The higher availability of the soluble components is due to the higher efficiency of the starch breakdown, as obtained with the higher WSI of LF. Due to increasing LF levels leading to higher WSI, the M75 formulation had the highest WSI in PBs.

A three‐way univariate ANOVA was performed to determine the main and interactive effects of legume flour level (LF), barrel temperature (BT), and feed moisture (FM) on the water solubility index (WSI) of the extruded samples (Appendix S3). Significant analysis showed that the whole model was significant (*F* (23, 24) = 12.081, *p* < 0.001), explaining a substantial percent of the variance in WSI (*R* ^2^ = 0.920, adjusted *R* ^2^ = 0.844). This shows how the factors and their interactions explained a substantial amount of the variation observed in the WSI values. The main effect of LF and BT was significant (*F* (3, 24) = 58.797, *p* < 0.001, partial *η* ^2^ = 0.880 and *F* (2, 24) = 8.586, *p* = 0.002, partial *η* ^2^ = 0.417, respectively), indicating that changes in legume flour amount and barrel temperature heavily affected WSI. The high content of LF increases the protein level in the extrudates, causing amino acid crosslinking and reducing its solubility (Maurya and Said 2014; Philipp et al. 2017). In the formulations made with 75% LF, increased BT provided higher WSI values. With increasing temperature, the gelatinization of starch increases, which results in more soluble starch and higher WSI. Increased WSI with temperature may be due to starch degradation and dextrinization during high‐shear extrusion, as reported by Singh et al. (2007) and Alam et al. (2016). This occurs because starch is dextrinized and depolymerized into smaller components, which are more soluble at higher temperatures (Ding et al. 2006). However, the main effect of FM appeared not to be statistically significant (*F* (1, 24) = 2.230, *p* = 0.148), thereby indicating that feed moisture, by itself, did not significantly affect WSI under the conditions tested. Highly significant two‐way interactions were observed between LF and BT (*F* (6, 24) = 3.006; *p* = 0.025; partial *η* ^2^ = 0.429), LF and FM (*F* (3, 24) = 5.862; *p* = 0.004; partial *η* ^2^ = 0.423), and BT and FM (F (2, 24) = 5.046; *p* = 0.015; partial *η* ^2^ = 0.296). In essence, these findings show that the effect of one variable on WSI depends upon the levels of the opposing interacting factor; this emphasizes the complexity involved in the extrusion process. Moreover, a highly significant three‐way interaction between LF, BT, and FM (*F* (6, 24) = 6.060; *p* = 0.001; partial *η* ^2^ = 0.602) was recorded, indicating a strong and nonadditive influence of all three processing parameters on the solubility characteristics of extruded samples.

The WAI is related to starch dispersion in excess water, enhanced by starch damage caused by gelatinization and fragmentation during extrusion that reduces amylose and amylopectin molecular weight. CPP exhibited the maximum WAI due to its high content of dietary fiber, with high water‐binding capacity (Ahmad et al. 2016; Kırbaş et al. 2019), while LF registered the lowest WAI, possibly due to the lesser amount of starch content limiting swelling and water retention, as well as high protein content hampering starch–water interaction. High WAI values indicate easier swelling and increased viscosity (Choi et al. 2012). The WAI of extruded products was analyzed through three‐way univariate analyses of variance to evaluate the effects of LF, BT, and FM, as well as their interactions (Appendix S3). The model is significant overall (*F* (23, 24) = 15.241, *p* < 0.001), and so the independent variables and their interaction account for most of the variation in WAI, *R* ^2^ = 0.936, Adjusted *R* ^2^ = 0.875. Among the main effects, LF significantly affected the WAI of extrusion products (*F* (3, 24) = 6.778, *p* = 0.002, *η* ^2^ = 0.459), which means that different levels of LF lead to statistically different WAI values of the extrusion products. Feed moisture also significantly affected WAI (*F* (1, 24) = 9.398, *p* = 0.005, *η* ^2^ = 0.281), but barrel temperature did not show any significant main effect (*F* (2, 24) = 0.062, *p* = 0.940) meaning that the factor hardly caused any change in the WAI under the tested conditions. Interaction effects in this respect suggest that two‐way interactions between LF and BT (*F* (6, 24) = 18.530, *p* < 0.001, *η* ^2^ = 0.822), LF and FM (*F* (3, 24) = 16.033, *p* < 0.001, *η* ^2^ = 0.667), and BT and FM (*F* (2, 24) = 11.827, *p* < 0.001, *η* ^2^ = 0.496) were all considered significant, meaning that these factors are combined and jointly act on WAI. Furthermore, most importantly, the interaction of the three in LF, BT, and FM was also significant (*F* (6, 24) = 22.960, *p* < 0.001, *η* ^2^ = 0.852), meaning that the influence of one factor on WAI depends highly on the level of the other two factors. WAI of extruded snacks was significantly affected by FM and LF (*p* ≤ 0.05), increasing as LF content increased and FM content decreased (Figure 2d). The observed WAI increase in the lower FM levels can be justified due to enhanced starch gelatinization and reduced plasticization that allow greater water uptake. Moreover, since LF is rich in protein and fiber, it becomes a natural agent for water binding and hence assures greater WAI (Bianchi and Simonato 2025). However, under certain conditions, protein–starch interactions can inhibit starch swelling and water uptake to reduce WAI (Zhang et al. 2025). These two opposing effects also highlight the factors of formulation and processing conditions in controlling the hydration behavior of starch–protein systems. Higher BD and larger particle size with increased CG result in reduced starch conversion and granule damage, hence reduced WAI values (Ding et al. 2006). Smaller‐sized starch particles increase the surface area during extrusion, and this increases shear‐induced fragmentation and dextrinization, allowing for better water absorption of the starches (Carvalho et al. 2010). Higher levels of FM were found to exhibit lower WAI, probably due to the molecular changes in the starch–protein matrix in LF at specific processing conditions.

The pasting properties of CG‐and LF‐enriched blends closely relate to their WAI and WSI behaviors. CG with high starch content showed continuous viscosity increases during pasting due to starch granule swelling and amylose leaching. According to the WAI and WSI parameters in their raw forms, it could be inferred that the granules remained intact, having low solubility. However, during extrusion, the CG blends showed a greater water absorption because of starch gelatinization compared with the LF blends, indicating that the LF blends absorbed less water due to the LF's high protein content that would compete with starch for water and eventually restrict the physical swelling of starch granules. This competition between protein and starch for water explains the reduced viscosity development found in the pasting analyses of LF‐enriched blends, in that proteins inhibit the leaching of amylose and form a network. In the context of the extruded snacks produced as a result of this study, higher values of WAI would be associated with chewier, denser textures brought about by enhanced water‐binding and reduced expansion. In comparison, lower values of WAI are associated with lighter, crisper textures needed in snack food formulations that are extruded.

The OAC is also in charge of regulating the structure, texture, and mouthfeel of food products produced using legume flours (Ahmad et al. 2016). OAC values for the LF, PF, CS, CPP, and their mixtures were determined. LF significantly affected the content at *p* ≤ 0.05. Since hydrophobic proteins are essential in oil absorption, a decrease in OAC with increased LF might be due to the higher content of hydrophilic proteins in LF, which absorb water rather than oil. Increased protein content, especially the presence of hydrophilic proteins, contributes to the lower OAC in LF. OAC values of various flours depend on different parameters like particle size, starch, protein content, and even the higher fiber content, as in carrot pomace powder (Drakos et al. 2017; Ahmad et al. 2016). In order to determine the raw materials' capacity to store fat before extrusion, OAC was only evaluated at the ingredient level in this investigation. Although the functional characteristics of various components might be compared thanks to this method, the degree of oil retention in the finished extruded products is not assessed. However, it is expected that extrudates with higher bulk density and lower porosity—resulting from high FM or LF levels—would exhibit lower oil absorption due to fewer capillary spaces. This assumption provides an indirect understanding of how extrusion conditions may influence oil retention. Fat‐binding behavior may be considerably changed by structural changes that occur during extrusion, such as matrix expansion and starch gelatinization. Therefore, to obtain a more thorough understanding of post‐extrusion lipid behavior and its impact on the mouthfeel, flavor release, and overall sensory perception, future research should consider assessing oil retention or total fat content in the extrudates.

### 3.7 SEM and EDX Analysis

**FIGURE 3:**
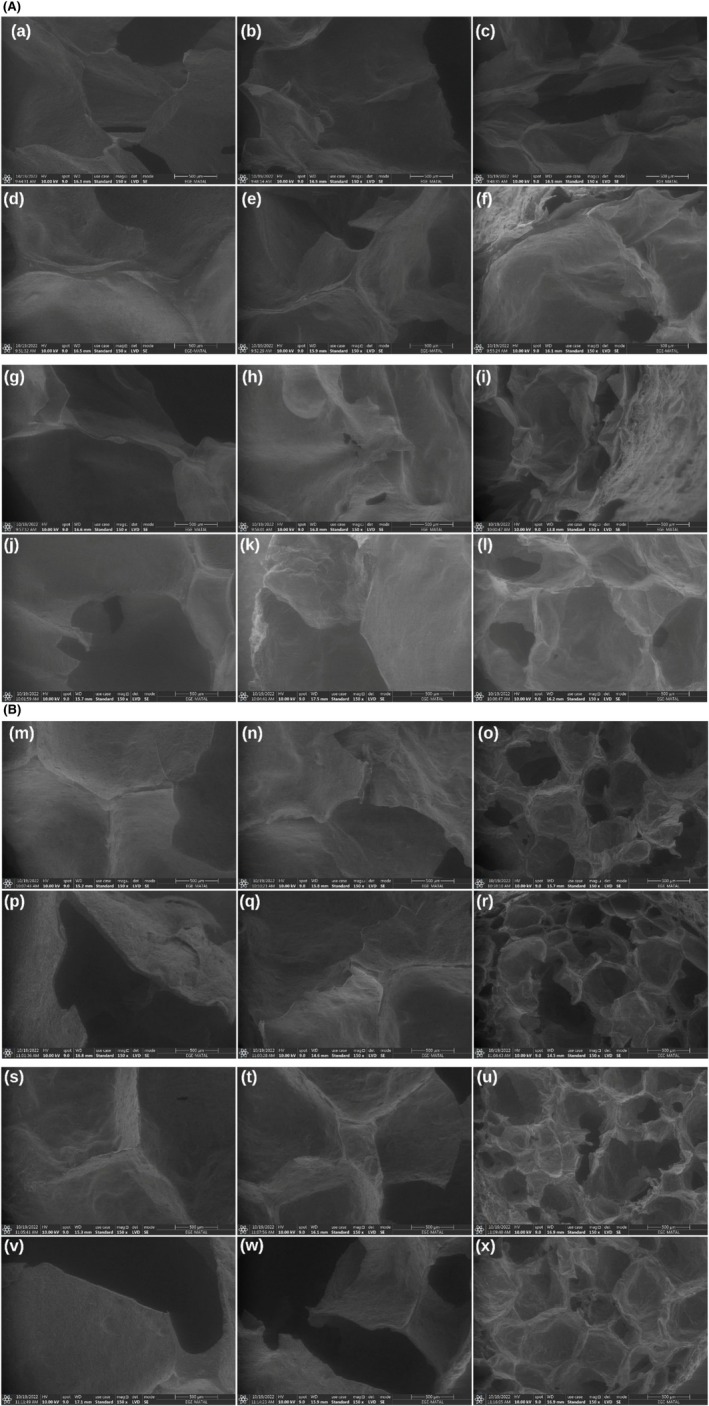
(A) SEM images of the extruded snacks (a) L0M16T135, (b) L0M16T150, (c) L0M16T165, (d) L0M18T135, (e) L0M18T150, (f) L0M18T165, (g) L25M16T135, (h) L25M16T150, (i) L25M16T165, (j) L25M18T135, (k) L25M18T150, (l) L25M18T165. (B) SEM images of the extruded snacks (m) L50M16T135, (n) L50M16T150, (o) L50M16T165, (p) L50M18T135, (q) L50M18T150, (r) L50M18T165, (s) L75M16T135, (t) L75M16T150, (u) L75M16T165, (v) L75M18T135, (w) L75M18T150, (x) L75M18T165. The coding of the samples was expressed to indicate LF, FM content, and extruder BT.

**FIGURE 4:**
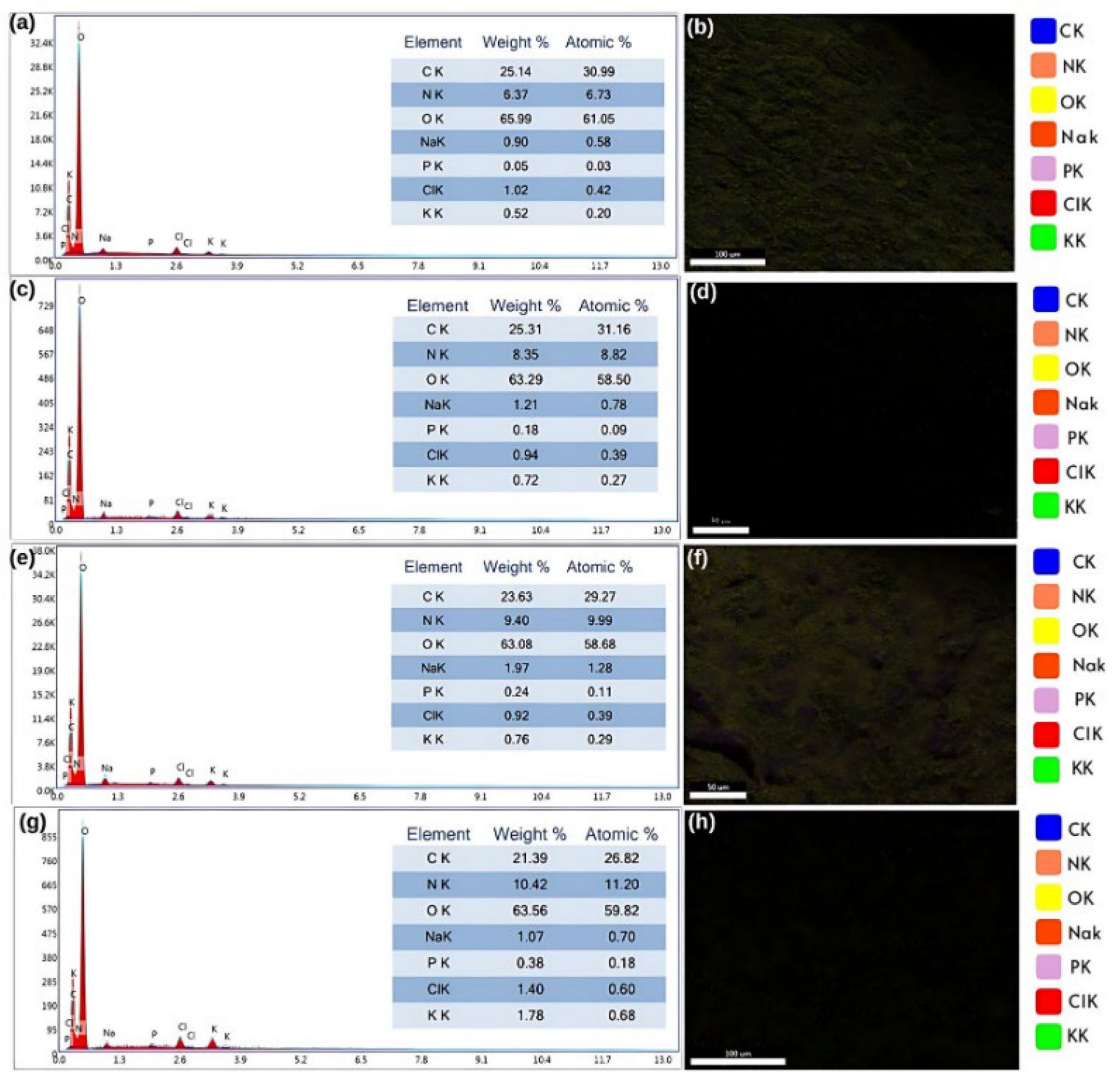
EDX images of extruded snacks (a) L0M16T135 of spectra showing elemental distribution, (b) L0M16T135 of colored maps by element, (c) L25M16T135 of spectra showing elemental distribution, (d) L25M16T135 of colored maps by element, (e) L50M16T135 of spectra showing elemental distribution, (f) L50M16T135 of colored maps by element, (g) L75M16T135 of spectra showing elemental distribution, (h) L75M16T135 of colored maps by element.

The morphological properties and element distribution of extruded snacks were determined using SEM and SEM‐EDX (Figures 3 and 4). The addition of LF to the formulation resulted in the formation of fibrous structures (Figure 3A‐a,B‐s). Morales et al. (2015) indicated that the total dietary fiber content of legumes is largely preserved after extrusion, and a transformation between soluble and insoluble dietary fibers may occur during the process. Similarly, it was noticed that with the increase in LF content, the fibrous structure became stronger, air bubbles became smaller, and a harder structure formed (Figure 3A‐i,B‐u). LF thickened the cell walls of extruded snacks and influenced airspace size. Lazou and Krokida (2010) observed that LF addition thickened the cell walls of ESs. Increasing FM content produced extruded snacks with cell walls of increasing thickness (Figure 3B‐n,q), likely due to its effect on starch gelatinization and matrix structure. Greater feed moisture is a plasticizer that reduces the shear intensity and allows for more regulated gelatinization, thereby creating a more dense and compact structure. Greater moisture also preserves starch granule integrity by preventing overfragmentation and producing thicker cell walls. On the other hand, increased BT was characterized by an increased amount of air cells and a reduction in cell wall thickness (Figure 3B‐s,t). This is because higher barrel temperature leads to rapid evaporation of moisture as the material exits the extruder, leading to a higher degree of expansion and the formation of more air bubbles. Overheating also degrades the starch–protein matrix, reducing structural strength and leading to thinner cell walls. The extruded snacks prepared with CG showed large‐sized air cells, thin walls, and weaker structures; the LF air cells had smaller sizes with an increased number of air bubbles and lower expansion. The extruded snacks had a denser and more compact structure as a result of LF's higher protein and dietary fiber content than CG's (Lazou and Krokida 2010). The SEM images revealed that with increasing LF content, the structures were denser and less expanded, perhaps due to the fact that with higher protein content, there was a restriction to starch expansion. These structural attributes correspond to the lower expansion ratios and greater hardness values of instrumental texture analysis. Conversely, samples with lower FM and intermediate BT contents had more open‐cell and porous structures, which corresponded to greater expansion and lower hardness. These results suggest the direct relationship of microstructural compactness with physical parameters, which has also been noted by Philipp et al. (2017).

SEM–EDX was used to examine how LF affected the element distribution in extruded snacks (Figure 4). Carbon and oxygen were the majority of elements present in all samples, as would be expected from the polymeric nature of the base matrix. Notably, nitrogen content was observed to enhance with higher levels of LF content, while carbon and oxygen contents gradually reduced. This is in accordance with the enhanced protein fraction in LF, considering the peptide bond nature of proteins and the resultant high nitrogen content. Such observations are in agreement with previous results in extruded food systems, where such elemental changes were likewise observed upon the addition of protein. For instance, Milani et al. (2024) pointed out increased nitrogen content in sesame cake‐containing extrudates due to its protein‐rich nature, while Guerrero et al. (2012) stressed structural and compositional alterations in soy protein‐based materials extruded in the presence of gelatin and sugars under low‐moisture extrusion. The current work's elemental trends are thus in agreement with established research and further attest to the addition of LF in the ES matrix.

### 3.8 Sensory Analysis

Sensory evaluation was conducted using 15 semitrained panel members to determine the sensory attributes of extruded snacks (Jeddou et al. 2017). Panel members rated the appearance, breakability, crispness, and hardness of the products on a nine‐point hedonic scale. Flavor was not examined in this study. The results showed that higher FM content increased breakability scores and decreased appearance and crispness scores (Table 3). This suggests that high moisture content negatively affects the textural attributes of ESs. Lower LF content was found to enhance appearance scores, which could indicate that lowering LF content improves the visual appeal of the product. These trends are consistent with those of previous studies on fiber‐enriched and pulse‐based extruded snacks. For example, Bisharat et al. (2013) reported that the addition of fiber‐rich by‐products (e.g., broccoli pomace) in extruded snacks reduced sensory crispness and acceptability. On the other hand, Alam et al. (2016) and Altan et al. (2008) observed that moderate inclusion of dietary fiber through lentils or tomato pomace held acceptable sensory qualities, especially when expansion was preserved. Our findings also agree with studies that reported a higher expansion ratio to be associated with improved sensory acceptance due to its role in enhancing crispness and reducing perceived hardness (Ding et al. 2006). Sensory values were evaluated alongside instrumental texture measurements (breakability, hardness) and color. The data show that consumer acceptability is increased in products with a higher expansion ratio, and higher FM content can have undesirable effects on good textural attributes. These comparisons indicate the necessity of determining the ratio of FM to LF since excessive moisture or fiber can undermine gluten‐free extruded food's structural and sensory quality. A Pearson correlation was used to investigate the relationship between the instrumental textural parameters and the sensory attributes. Hardness and perceived hardness showed a weak correlation (*r* = 0.13, *p* < 0.05), indicating a limited association between the two measurements despite being statistically significant. However, crispiness and perceived crispiness showed a moderate positive correlation (*r* = 0.68, *p* < 0.05), suggesting that the sensory perception of crispiness was consistent with the instrumental data.

### 3.9 Multivariate Statistical Analysis

**FIGURE 5:**
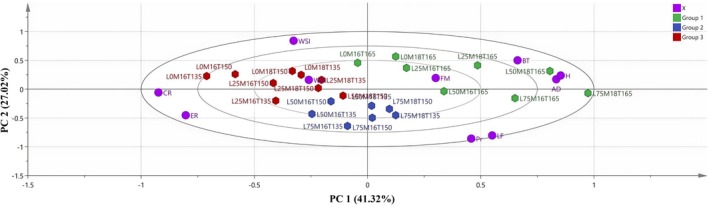
Biplot of principal component analysis for extruded snacks (AD, apparent density; BT, barrel temperature; CR, crispiness; ER, expansion ratio; FM, feed moisture; H, hardness; LF, lentil flour content; Pr, protein content; WAI, water absorption index; WSI, water solubility index). The coding of the samples was expressed to indicate LF, FM content, and extruder BT.

LF content, FM, and BT were analyzed by multivariate statistical analysis to find the impact on the physicochemical and functional properties of the ESs. The interrelationship among the variables was analyzed by PCA and HCPC. The biplot of PCA loadings and scores is represented in Figure 5 (*R* ^2^ = 0.905). From that PCA model, the variance accounted for in the total dataset is 90.49%. Thus, in this result, PC1 explained about 41.32% of the variance (4.13 was the eigenvalue), while PC2 accounted for about 27.02% of the variation in this dataset, with a corresponding eigenvalue of about 2.70. The third, PC3, accounted for 13.64% with an eigenvalue of 1.36, while the fourth, PC4, explained 8.50% with an eigenvalue of 0.85. In the biplot, the extruded snacks fell into three clusters, and no single independent variable was responsible for categorization. PC1 was positively related to H and AD, whereas ER and CR were placed on the negative side, indicating that these properties were inversely related. Most of the extruded snacks produced at the maximum settings of BT fell in the positive section of PC1, which indicates that with an increase in processing temperature, hardness, and density increase, and expansion and crispness decline. PC2, on the other hand, was negatively associated with LF and protein content and helped distinguish samples based on compositional differences due to these parameters. Furthermore, PC1 shows WAI to be inversely related to FM, indicating that higher moisture contents may act to limit starch gelatinization and eventual water absorption.

HCPC further supported the PCA patterns. The clustering set apart the extruded snack types into three main groups, considering formulation‐ and process‐variable effects together. Group 1 (green) included samples subjected to higher barrel temperatures and also a moderate‐to‐high level of lentil flour, demonstrating higher H values with AD, which suggests the product is more compact and more rigid in structure. Group 2 (blue) featured samples cooked under intermediate settings in both formulation and process conditions and held intermediate levels of the physicochemical parameters evaluated. On the other hand, Group 3 (red) comprised samples produced with either low BT or LF levels and were characterized by high ER and CR values, ideal for a light and aerated snack texture. Such findings have also been evidenced in earlier literature, stating that a low protein and fiber presence coupled with moderate thermal presence aids starch expansion, thus conferring crisp and porous structures in extrudates (Lazou et al. 2007; Devi et al. 2013; Giannini et al. 2013).

## 4 Conclusion

The conducted study demonstrated the production of new extruded snacks containing LF. The study highlights the substantial influence of ingredient blending and extrusion parameters on the nutritional profile, sensory attributes, and structural properties of the product. The outcomes displayed that the variables investigated have a significant impact on the characteristics of extruded snacks. In particular, increasing LF enhanced the protein content in snacks, thereby yielding more compact, harder snacks with lower expansion ratios and a denser texture. High FM resulted in higher water retention and, therefore, a more extensive cell wall and denser, firmer texture. In contrast, higher BT expanded the air bubbles and thinned the cell walls, giving a less dense structure. Moreover, LF's substantial protein content reduced its solubility and affected its pasting properties. Extrusion conditions affected not only the texture and color features of the snacks but also interacted synergistically to influence their physical, nutritional, and sensory properties. The present study also contributes to the literature on gluten‐free extrusion and suggests an innovative way of incorporating lentil flour into the expanded snack matrix to assist in complementing its functional and sensory attributes. These findings reflect the conflict between protein enrichment and sensory attributes of gluten‐free extruded snacks. Enrichment with LF improves nutritional quality but is detrimental to texture and expansion. However, some limitations exist. An experimental procedure took place under certain extrusion conditions, with the screw speed set as a fixed parameter. Because of this, the results might not fully apply to other extrusion methods. Additionally, a larger sensory panel, along with an analysis of consumer acceptance, could have provided more insight into the market potential of these snacks. This research provides insights into the development of healthier snacks with favorable texture and structure, offering an improved alternative to current gluten‐free snacks by utilizing extrusion technology.

Based on the experimental results, the combination of LF, FM, and BT that resulted in the most favorable textural and sensory characteristics was 25% LF, 16% FM, and 150°C BT. This condition resulted in an optimal combination of maximum expansion ratio, lower apparent density, intermediate hardness, and maximum crispness, along with improved nutritional quality with higher protein content. This mixture is suggested to be the most appropriate for preparing gluten‐free extruded snacks with enhanced consumer acceptability and structural characteristics. In the future, it will be important to look into pretreatments of lentil flour that reduce the loss of expansion or blending with other proteins or starches that could help with texture loss. In addition, microstructural studies and molecular analysis of starch–protein interactions may provide a better understanding and allow for greater control over the process.

## Author Contributions

**Buse Ozlem Esen:** conceptualization (equal), data curation (equal), formal analysis (equal), funding acquisition (equal), investigation (equal), methodology (equal), validation (equal), visualization (equal), writing – original draft (equal), writing – review and editing (equal). **Neslihan Bozdogan:** conceptualization (equal), data curation (equal), formal analysis (equal), investigation (equal), methodology (equal), software (equal), supervision (equal), validation (equal), visualization (equal), writing – original draft (equal), writing – review and editing (equal). **Lara Mina Kutlar:** data curation (equal), formal analysis (equal), investigation (equal), methodology (equal), validation (equal), writing – original draft (equal). **Seher Kumcuoglu:** conceptualization (equal), funding acquisition (equal), project administration (equal), resources (equal), supervision (equal), writing – review and editing (equal).

## Conflicts of Interest

The authors declare no conflicts of interest.

## Supporting information


Appendix S1.

Appendix S2.

Appendix S3.

## Data Availability

Data will be made available upon request.
