# Supplementary material for: Development of Gluten‐Free Extruded Snack Containing Lentil Flour and Evaluation of Extrusion Process Conditions on Quality Properties
Source: Food Sci Nutr. 2025 Jul 28;13(8):e70663. doi: 10.1002/fsn3.70663 (PMC12301571; doi:10.1002/fsn3.70663)
Supplement: Supplementary file 1 — Appendix S1. [file FSN3-13-e70663-s001.docx]

**
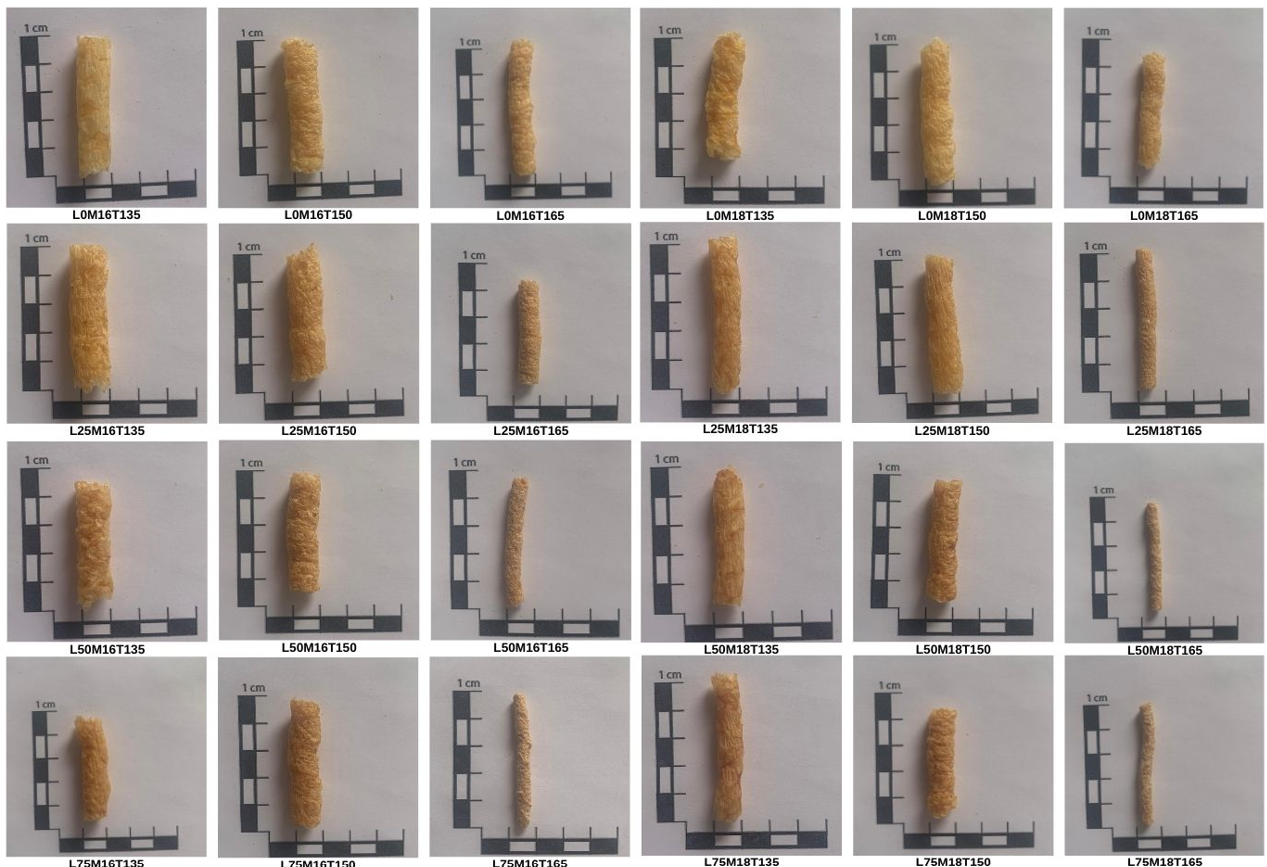
Supplementary 1.** The coding of the samples was expressed to indicate LF, FM content, and extruder BT. The numbers after the letter L indicate the LF ratio, the numbers after the letter M indicate the FM ratio and the numbers after the letter T indicate the extruder BT. For example, code L75M16T165 indicates a product produced at 75% LF, 16% FM, and an extruder BT of 165 °C. Values with similar superscripts in the column do not differ significantly (p < 0.05).
