# Supplementary material for: Development of Gluten‐Free Extruded Snack Containing Lentil Flour and Evaluation of Extrusion Process Conditions on Quality Properties
Source: Food Sci Nutr. 2025 Jul 28;13(8):e70663. doi: 10.1002/fsn3.70663 (PMC12301571; doi:10.1002/fsn3.70663)
Supplement: Supplementary file 2 — Appendix S2. [file FSN3-13-e70663-s003.docx]

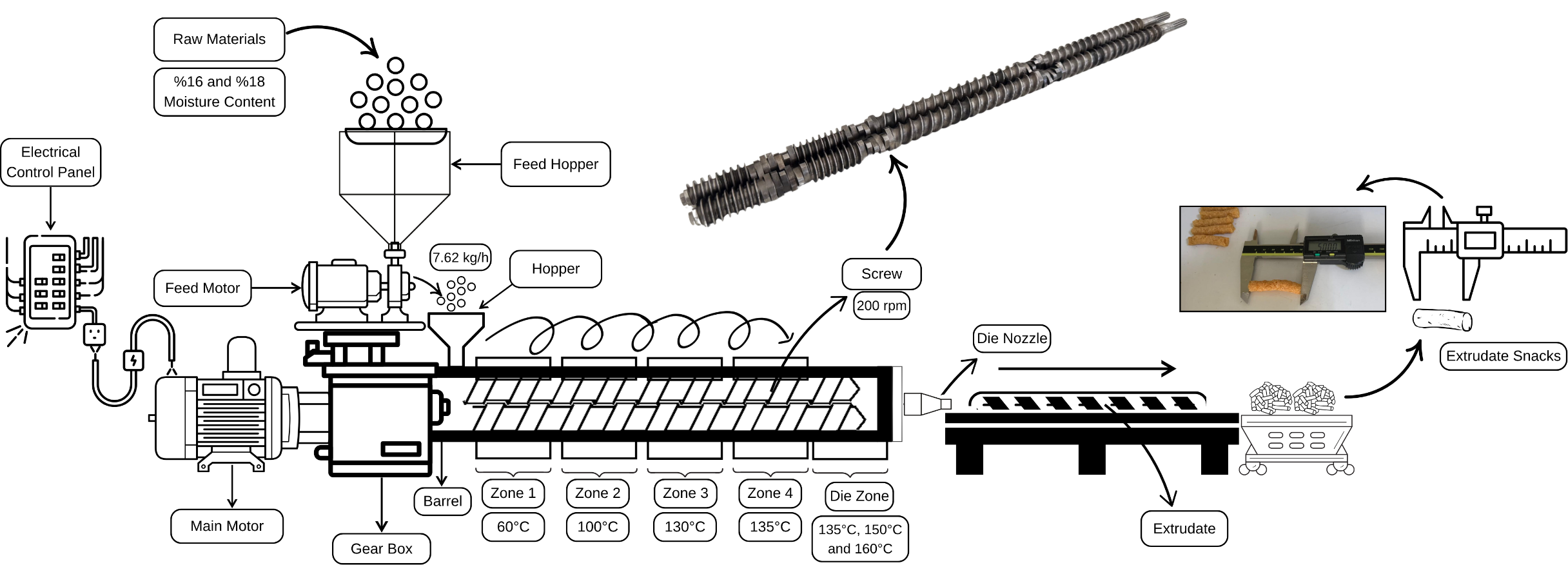


**Supplementary 2.** Schematic representation of the twin-screw extrusion system used in the production of gluten-free extruded snacks. The diagram illustrates the screw configuration, barrel zones, temperature control system, and die design.
