# Supplementary material for: Development of Gluten‐Free Extruded Snack Containing Lentil Flour and Evaluation of Extrusion Process Conditions on Quality Properties
Source: Food Sci Nutr. 2025 Jul 28;13(8):e70663. doi: 10.1002/fsn3.70663 (PMC12301571; doi:10.1002/fsn3.70663)
Supplement: Supplementary file 3 — Appendix S3. [file FSN3-13-e70663-s002.docx]

### **Supplemantary Material 3.** Effects of BT, FM, and LF on physicochemical properties (Tests of Between-Subjects Effects (ANOVA Results))

| **Properties** | **Source** | **df** | **F** | **Sig.** | **Partial η²** |
| --- | --- | --- | --- | --- | --- |
| **WAI** | LF | 3, 24 | 6.778 | 0.002 | 0.459 |
|  | BT | 2, 24 | 0.062 | 0.940 | 0.005 |
|  | FM | 1, 24 | 9.398 | 0.005 | 0.281 |
|  | LF × BT | 6, 24 | 18.530 | <0.001 | 0.822 |
|  | LF × FM | 3, 24 | 16.033 | <0.001 | 0.667 |
|  | BT × FM | 2, 24 | 11.827 | <0.001 | 0.496 |
|  | LF × BT × FM | 6, 24 | 22.960 | <0.001 | 0.852 |
| **WSI** | LF | 3, 24 | 58.797 | < .001 | 0.880 |
|  | BT | 2, 24 | 8.586 | 0.002 | 0.417 |
|  | FM | 1, 24 | 2.230 | 0.148 | 0.085 |
|  | LF × BT | 6, 24 | 3.006 | 0.025 | 0.429 |
|  | LF × FM | 3, 24 | 5.862 | 0.004 | 0.423 |
|  | BT × FM | 2, 24 | 5.046 | 0.015 | 0.296 |
|  | LF × BT × FM | 6, 24 | 6.060 | 0.001 | 0.602 |
| **Protein** | LF | 3, 24 | 3455.589 | < .001 | 0.998 |
|  | BT | 2, 24 | 39.668 | < .001 | 0.768 |
|  | FM | 1, 24 | 33.994 | < .001 | 0.586 |
|  | LF × BT | 6, 24 | 2.110 | 0.090 | 0.345 |
|  | LF × FM | 3, 24 | 5.306 | 0.006 | 0.399 |
|  | BT × FM | 2, 24 | 2.197 | 0.133 | 0.155 |
|  | LF × BT × FM | 6, 24 | 1.089 | 0.397 | 0.214 |
| **Crispiness** | LF | 3, 24 | 358.581 | <0 .001 | 0.884 |
|  | BT | 2, 24 | 1321.192 | <0 .001 | 0.949 |
|  | FM | 1, 24 | 172.664 | <0 .001 | 0.550 |
|  | LF × BT | 6, 24 | 10.292 | <0 .001 | 0.305 |
|  | LF × FM | 3, 24 | 18.067 | <0 .001 | 0.278 |
|  | BT × FM | 2, 24 | 16.485 | <0 .001 | 0.190 |
|  | LF × BT × FM | 6, 24 | 10.723 | <0 .001 | 0.313 |
| **Hardness** | LF | 3, 24 | 103.190 | <0.001 | 0.812 |
|  | BT | 2, 24 | 889.220 | <0.001 | 0.914 |
|  | FM | 1, 24 | 683.784 | <0.001 | 0.804 |
|  | LF × BT | 6, 24 | 17.809 | <0.001 | 0.348 |
|  | LF × FM | 3, 24 | 8.226 | <0.001 | 0.129 |
|  | BT × FM | 2, 24 | 171.813 | < 0.001 | 0.673 |
|  | LF × BT × FM | 6, 24 | 5.027 | 0.008 | 0.057 |
| **Expansion Ratio** | LF | 3, 24 | 210.467 | <0.001 | 0.745 |
|  | BT | 2, 24 | 2353.336 | <0.001 | 0.956 |
|  | FM | 1, 24 | 711.839 | <0.001 | 0.767 |
|  | LF × BT | 6, 24 | 41.614 | <0.001 | 0.536 |
|  | LF × FM | 3, 24 | 37.250 | <0.001 | 0.341 |
|  | BT × FM | 2, 24 | 18.541 | <0.001 | 0.147 |
|  | LF × BT × FM | 6, 24 | 43.485 | <0.001 | 0.547 |
| **Apparent Density** | LF | 3, 24 | 468.413 | <0.001 | 0.955 |
|  | BT | 2, 24 | 4160.447 | <0.001 | 0.984 |
|  | FM | 1, 24 | 887.144 | <0.001 | 0.870 |
|  | LF × BT | 6, 24 | 492.997 | <0.001 | 0.957 |
|  | LF × FM | 3, 24 | 19.059 | <0.001 | 0.301 |
|  | BT × FM | 2, 24 | 234.146 | <0.001 | 0.779 |
|  | LF × BT × FM | 6, 24 | 29.986 | <0.001 | 0.474 |

*df = degrees of freedom; Partial η² = effect size.

**LF indicates the lentil flour, FM indicates the feed moisture, and BT indicates the barrel tempreture of the extruder.
